# Supplementary material for: Development of SNP Set for the Marker-Assisted Selection of Guar (Cyamopsis tetragonoloba (L.) Taub.) Based on a Custom Reference Genome Assembly
Source: Plants (Basel). 2021 Sep 30;10(10):2063. doi: 10.3390/plants10102063 (PMC8539970; doi:10.3390/plants10102063)
Supplement: Supplementary file 1 [file plants-10-02063-s001.zip › figures/Supplementary Materials Figure S6.pdf]

Supplementary Materials Figure S5. Validation of SNPs and deletions associated with early maturation by PCR and Sanger sequencing

Primer list

Locus	Forward	Revers	Expected size
contig_59:14389378 (gu59)	AGAACCACACATTCTTGACACTA	TGGTGGACATCCCTCTCTAA	418
contig_229:330388 (gu229)	CCAAGTGTCATTCTCCGTTAGT	GGTCTGATCTGTCGTGCATATAG	500
contig_88:4931265 (gu88)	GCGTGGAGTTTAGTGGACTATG	GTGCTCTCTCTAAGGCTGAAAC	347
contig_182:841660 (gu182)	GACTCTCCATCATAGGGTTTACTT	AGTCCTGTCTCTCCTGGTAAT	550
contig_37:351180 (gu37)	CGAACAAACGGTCAGCAAATAA	AAATGAAGCACGTGAAGCTAATC	553
contig_188:3026232 (gu188)	GCCAGTCCAATAGGCTAACA	CCTGCCTGACTCAGATAAGAAC	638

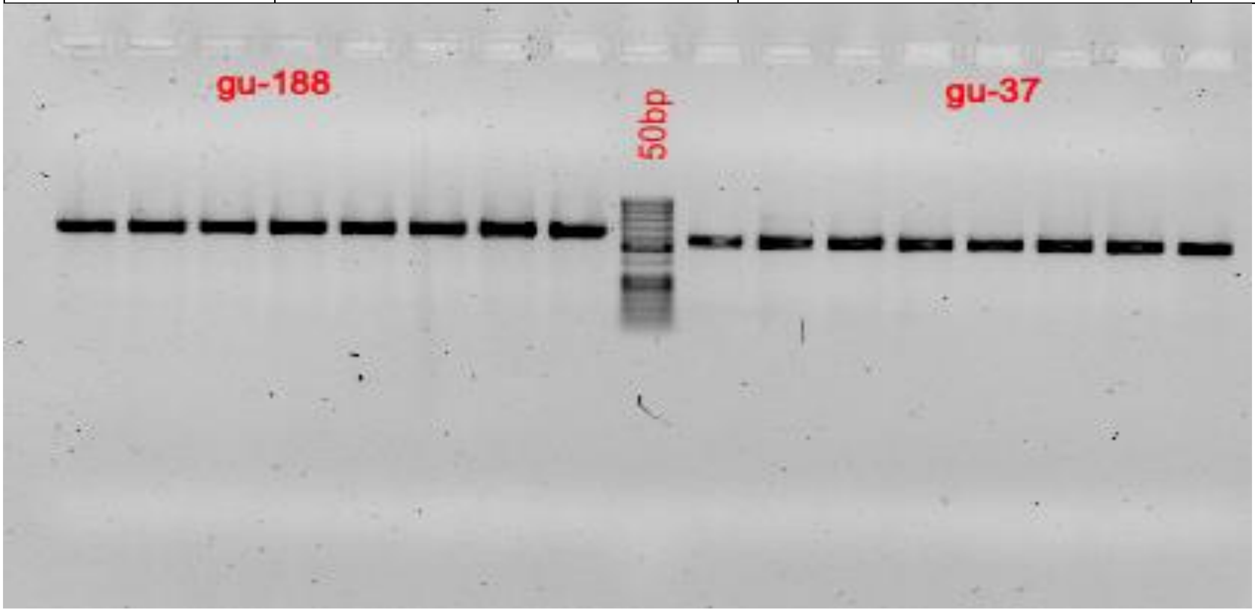

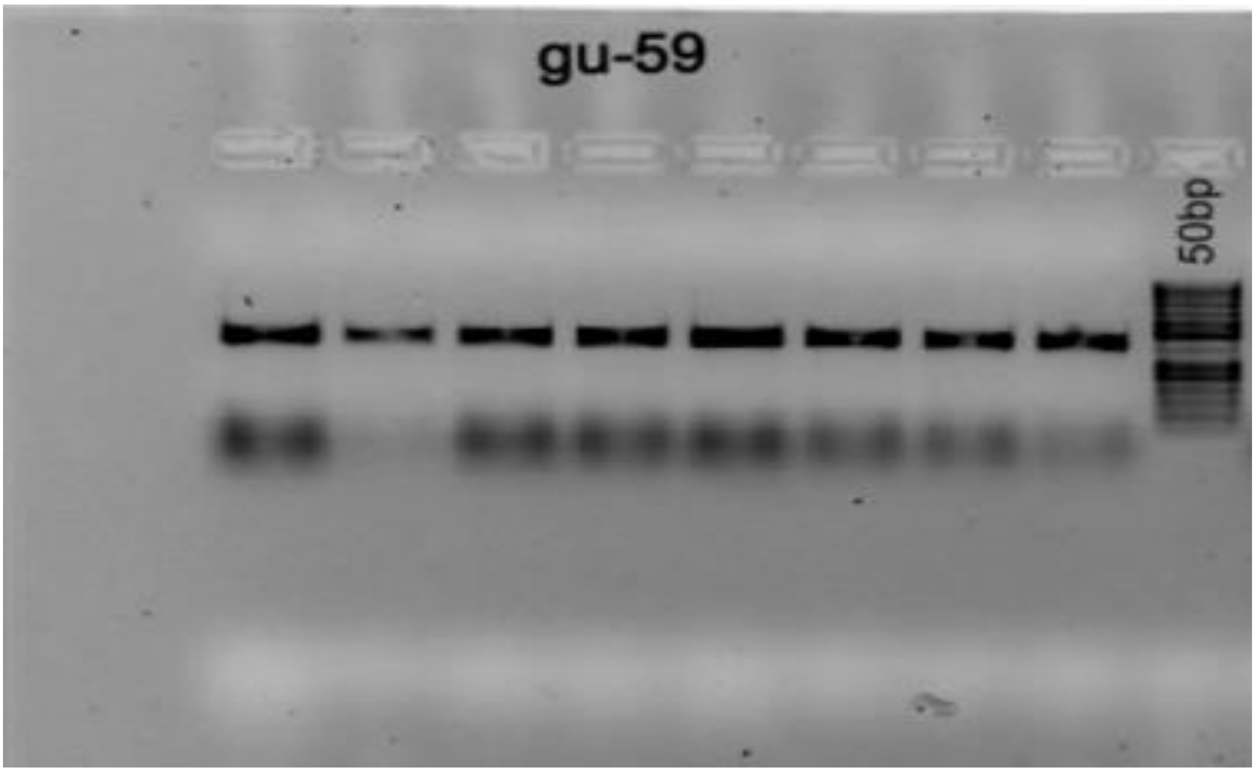

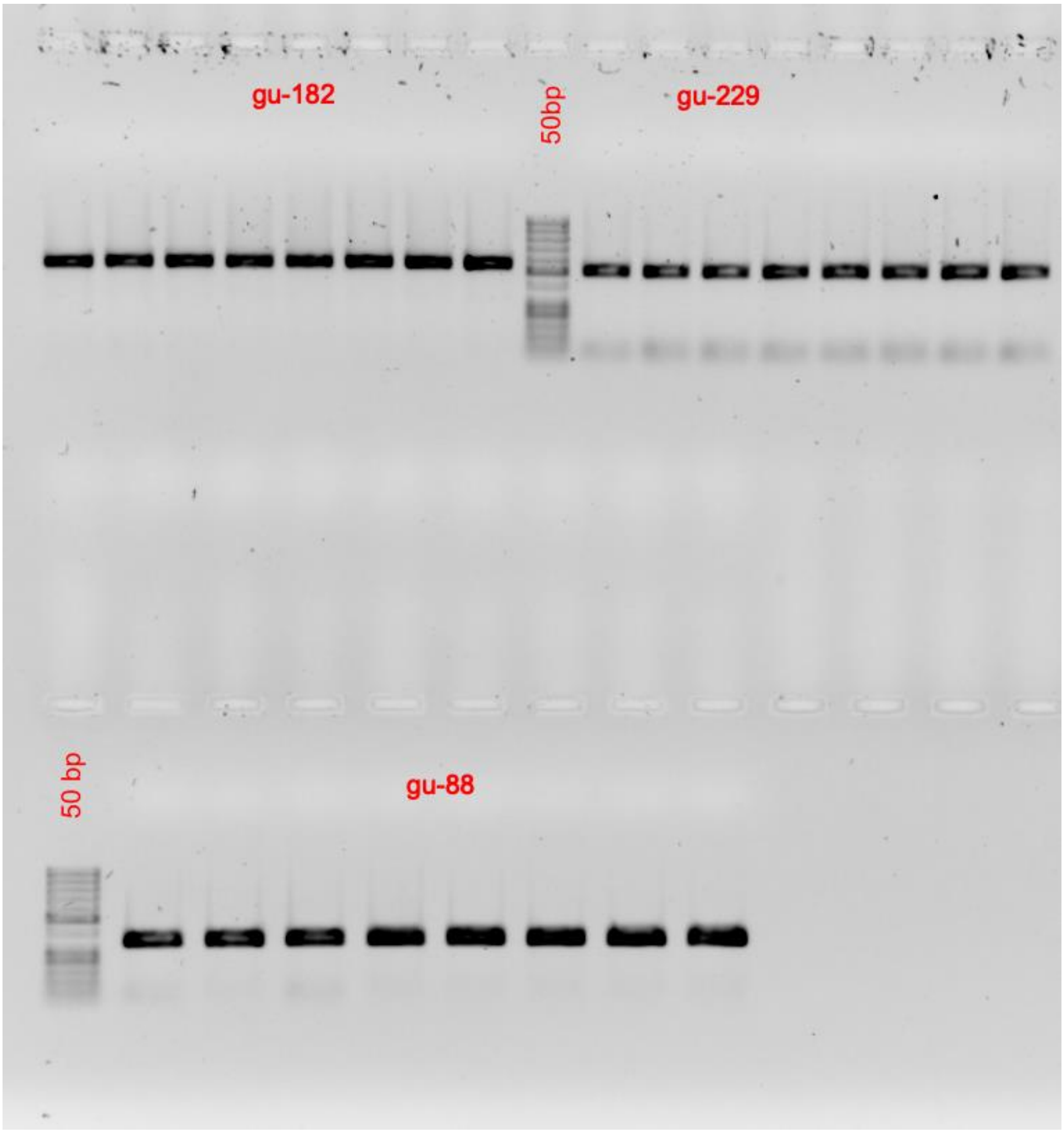
gu-182
50bp
gu-229
50 bp
gu-88

```
                 20                  40                  60
                  |                   |                   |
37-contig AATAAACGAT CCGTAAACAA ATGGTCAGTG AATAAACGGT CAGTGAATAA GAGTCAGTGA 60
      s73 AATAAACGAT CCGTAAACAA ATGGTCAGTG AATAAACGGT CAGTGAATAA GAGTCAGTGA 60
    s73-r AATAAACGAT CCGTAAACAA ATGGTCAGTG AATAAACGGT CAGTGAATAA GAGTCAGTGA 60
      s81 AATAAACGAT CCGTAAACAA ATGGTCAGTG AATAAACGGT CAGTGAATAA GAGTCAGTGA 60
    s81-r AATAAACGAT CCGTAAACAA ATGGTCAGTG AATAAACGGT CAGTGAATAA GAGTCAGTGA 60
      s59 AATAAACGAT CCGTAAACAA ATGGTCAGTG AATAAACGGT CAGTGAATAA GAGTCAGTGA 60
    s59-r AATAAACGAT CCGTAAACAA ATGGTCAGTG AATAAACGGT CAGTGAATAA GAGTCAGTGA 60
      s51 AATAAACGAT CCGTAAACAA ATGGTCAGTG AATAAACGGT CAGTGAATAA GAGTCAGTGA 60
    s51-r AATAAACGAT CCGTAAACAA ATGGTCAGTG AATAAACGGT CAGTGAATAA GAGTCAGTGA 60
      s36 AATAAACGAT CCGTAAACAA ATGGTCAGTG AATAAACGGT CAGTGAATAA GAGTCAGTGA 60
    s36-r AATAAACGAT CCGTAAACAA ATGGTCAGTG AATAAACGGT CAGTGAATAA GAGTCAGTGA 60
      s33 AATAAACGAT CCGTAAACAA ATGGTCAGTG AATAAACGGT CAGTGAATAA GAGTCAGTGA 60
    s33-r AATAAACGAT CCGTAAACAA ATGGTCAGTG AATAAACGGT CAGTGAATAA GAGTCAGTGA 60
      s26 AATAAACGAT CCGTAAACAA ATGGTCAGTG AATAAACGGT CAGTGAATAA GAGTCAGTGA 60
    s26-r AATAAACGAT CCGTAAACAA ATGGTCAGTG AATAAACGGT CAGTGAATAA GAGTCAGTGA 60
      s19 AATAAACGAT CCGTAAACAA ATGGTCAGTG AATAAACGGT CAGTGAATAA GGGTCAGTGA 60
    s19-r AATAAACGAT CCGTAAACAA ATGGTCAGTG AATAAACGGT CAGTGAATAA GGGTCAGTGA 60
     s110 AATAAACGAT CCGTAAACAA ATGGTCAGTG AATAAACGGT CAGTGAATAA GGGTCAGTGA 60
   s110-r AATAAACGAT CCGTAAACAA ATGGTCAGTG AATAAACGGT CAGTGAATAA GGGTCAGTGA 60
     s170 AATAAACGAT CCGTAAACAA ATGGTCAGTG AATAAACGGT CAGTGAATAA GGGTCAGTGA 60
   s170-r AATAAACGAT CCGTAAACAA ATGGTCAGTG AATAAACGGT CAGTGAATAA GGGTCAGTGA 60
     s109 AATAAACGAT CCGTAAACAA ATGGTCAGTG AATAAACGGT CAGTGAATAA GGGTCAGTGA 60
   s109-r AATAAACGAT CCGTAAACAA ATGGTCAGTG AATAAACGGT CAGTGAATAA GGGTCAGTGA 60
     s168 AATAAACGAT CCGTAAACAA ATGGTCAGTG AATAAACGGT CAGTGAATAA GGGTCAGTGA 60
   s168-r AATAAACGAT CCGTAAACAA ATGGTCAGTG AATAAACGGT CAGTGAATAA GGGTCAGTGA 60
Consensus AATAAACGAT CCGTAAACAA ATGGTCAGTG AATAAACGGT CAGTGAATAA GAGTCAGTGA
     100%
Conservation
       0%

                 80                  100                 120
                  |                   |                   |
37-contig GCAAGGGTCA GGCGTGAGAA CAATAACTCA TAGAGCAAGC ATAATCCAAA TAATGACATG 120
      s73 GCAAGGGTCA GGCGTGAGAA CAATAACTCA TAGAGCAAGC ATAATCCAAA TAATGACATG 120
    s73-r GCAAGGGTCA GGCGTGAGAA CAATAACTCA TAGAGCAAGC ATAATCCAAA TAATGACATG 120
      s81 GCAAGGGTCA GGCGTGAGAA CAATAACTCA TAGAGCAAGC ATAATCCAAA TAATGACATG 120
    s81-r GCAAGGGTCA GGCGTGAGAA CAATAACTCA TAGAGCAAGC ATAATCCAAA TAATGACATG 120
      s59 GCAAGGGTCA GGCGTGAGAA CAATAACTCA TAGAGCAAGC ATAATCCAAA TAATGACATG 120
    s59-r GCAAGGGTCA GGCGTGAGAA CAATAACTCA TAGAGCAAGC ATAATCCAAA TAATGACATG 120
      s51 GCAAGGGTCA GGCGTGAGAA CAATAACTCA TAGAGCAAGC ATAATCCAAA TAATGACATG 120
    s51-r GCAAGGGTCA GGCGTGAGAA CAATAACTCA TAGAGCAAGC ATAATCCAAA TAATGACATG 120
      s36 GCAAGGGTCA GGCGTGAGAA CAATAACTCA TAGAGCAAGC ATAATCCAAA TAATGACATG 120
    s36-r GCAAGGGTCA GGCGTGAGAA CAATAACTCA TAGAGCAAGC ATAATCCAAA TAATGACATG 120
      s33 GCAAGGGTCA GGCGTGAGAA CAATAACTCA TAGAGCAAGC ATAATCCAAA TAATGACATG 120
    s33-r GCAAGGGTCA GGCGTGAGAA CAATAACTCA TAGAGCAAGC ATAATCCAAA TAATGACATG 120
      s26 GCAAGGGTCA GGCGTGAGAA CAATAACTCA TAGAGCAAGC ATAATCCAAA TAATGACATG 120
    s26-r GCAAGGGTCA GGCGTGAGAA CAATAACTCA TAGAGCAAGC ATAATCCAAA TAATGACATG 120
      s19 GCAAGGGTCA GGCGTGAGAA CAATAACTCA TAGAGCAAGC ATAATCCAAA TAATGACATG 120
    s19-r GCAAGGGTCA GGCGTGAGAA CAATAACTCA TAGAGCAAGC ATAATCCAAA TAATGACATG 120
     s110 GCAAGGGTCA GGCGTGAGAA CAATAACTCA TAGAGCAAGC ATAATCCAAA TAATGACATG 120
   s110-r GCAAGGGTCA GGCGTGAGAA CAATAACTCA TAGAGCAAGC ATAATCCAAA TAATGACATG 120
     s170 GCAAGGGTCA GGCGTGAGAA CAATAACTCA TAGAGCAAGC ATAATCCAAA TAATGACATG 120
   s170-r GCAAGGGTCA GGCGTGAGAA CAATAACTCA TAGAGCAAGC ATAATCCAAA TAATGACATG 120
     s109 GCAAGGGTCA GGCGTGAGAA CAATAACTCA TAGAGCAAGC ATAATCCAAA TAATGACATG 120
   s109-r GCAAGGGTCA GGCGTGAGAA CAATAACTCA TAGAGCAAGC ATAATCCAAA TAATGACATG 120
     s168 GCAAGGGTCA GGCGTGAGAA CAATAACTCA TAGAGCAAGC ATAATCCAAA TAATGACATG 120
   s168-r GCAAGGGTCA GGCGTGAGAA CAATAACTCA TAGAGCAAGC ATAATCCAAA TAATGACATG 120
Consensus GCAAGGGTCA GGCGTGAGAA CAATAACTCA TAGAGCAAGC ATAATCCAAA TAATGACATG
     100%
Conservation
       0%

                 140                 160                 180
                  |                   |                   |
37-contig AGTTAAAAGA GCTTCTTATT CCTCCTCTAC TACAAAGGCG CTTTGTCAAG GTATGAACAT 180
      s73 AGTTAAAAGA GCTTCTTATT CCTCCTCTAC TACAAAGGCG CTTTGTCAAG GTATGAACAT 180
    s73-r AGTTAAAAGA GCTTCTTATT CCTCCTCTAC TACAAAGGCG CTTTGTCAAG GTATGAACAT 180
      s81 AGTTAAAAGA GCTTCTTATT CCTCCTCTAC TACAAAGGCG CTTTGTCAAG GTATGAACAT 180
    s81-r AGTTAAAAGA GCTTCTTATT CCTCCTCTAC TACAAAGGCG CTTTGTCAAG GTATGAACAT 180
      s59 AGTTAAAAGA GCTTCTTATT CCTCCTCTAC TACAAAGGCG CTTTGTCAAG GTATGAACAT 180
    s59-r AGTTAAAAGA GCTTCTTATT CCTCCTCTAC TACAAAGGCG CTTTGTCAAG GTATGAACAT 180
      s51 AGTTAAAAGA GCTTCTTATT CCTCCTCTAC TACAAAGGCG CTTTGTCAAG GTATGAACAT 180
    s51-r AGTTAAAAGA GCTTCTTATT CCTCCTCTAC TACAAAGGCG CTTTGTCAAG GTATGAACAT 180
      s36 AGTTAAAAGA GCTTCTTATT CCTCCTCTAC TACAAAGGCG CTTTGTCAAG GTATGAACAT 180
    s36-r AGTTAAAAGA GCTTCTTATT CCTCCTCTAC TACAAAGGCG CTTTGTCAAG GTATGAACAT 180
      s33 AGTTAAAAGA GCTTCTTATT CCTCCTCTAC TACAAAGGCG CTTTGTCAAG GTATGAACAT 180
    s33-r AGTTAAAAGA GCTTCTTATT CCTCCTCTAC TACAAAGGCG CTTTGTCAAG GTATGAACAT 180
      s26 AGTTAAAAGA GCTTCTTATT CCTCCTCTAC TACAAAGGCG CTTTGTCAAG GTATGAACAT 180
    s26-r AGTTAAAAGA GCTTCTTATT CCTCCTCTAC TACAAAGGCG CTTTGTCAAG GTATGAACAT 180
      s19 AGTTAAAAGA GCTTCTTATT CCTCCTCTAC TACAAAGGCG CTTTGTCAAG GTATGAACAT 180
    s19-r AGTTAAAAGA GCTTCTTATT CCTCCTCTAC TACAAAGGCG CTTTGTCAAG GTATGAACAT 180
     s110 AGTTAAAAGA GCTTCTTATT CCTCCTCTAC TGCAAAGGCG CTTTGTCAAG GTATGAACAT 180
   s110-r AGTTAAAAGA GCTTCTTATT CCTCCTCTAC TGCAAAGGCG CTTTGTCAAG GTATGAACAT 180
     s170 AGTTAAAAGA GCTTCTTATT CCTCCTCTAC TGCAAAGGCG CTTTGTCAAG GTATGAACAT 180
   s170-r AGTTAAAAGA GCTTCTTATT CCTCCTCTAC TGCAAAGGCG CTTTGTCAAG GTATGAACAT 180
     s109 AGTTAAAAGA GCTTCTTATT CCTCCTCTAC TGCAAAGGCG CTTTGTCAAG GTATGAACAT 180
   s109-r AGTTAAAAGA GCTTCTTATT CCTCCTCTAC TGCAAAGGCG CTTTGTCAAG GTATGAACAT 180
     s168 AGTTAAAAGA GCTTCTTATT CCTCCTCTAC TGCAAAGGCG CTTTGTCAAG GTATGAACAT 180
   s168-r AGTTAAAAGA GCTTCTTATT CCTCCTCTAC TGCAAAGGCG CTTTGTCAAG GTATGAACAT 180
Consensus AGTTAAAAGA GCTTCTTATT CCTCCTCTAC TACAAAGGCG CTTTGTCAAG GTATGAACAT
     100%
Conservation
       0%
```

Legend:

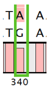
- target SNV

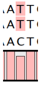
- non-target SNVs

```
                                     200                 220                 240
                                       |                   |                   |
37-contig  CTTGCATGCA TCATTCATTC AAAGCCTTCC AAGCCACGAG ACCCATTTAG TTCCCAAGCC 240
      s73  CTTGCATGCA TCATTCATTC AAAGCCTTCC AAGCCACGAG ACCCATTTAG TTCCCAAGCC 240
    s73-r  CTTGCATGCA TCATTCATTC AAAGCCTTCC AAGCCACGAG ACCCATTTAG TTCCCAAGCC 240
      s81  CTTGCATGCA TCATTCATTC AAAGCCTTCC AAGCCACGAG ACCCATTTAG TTCCCAAGCC 240
    s81-r  CTTGCATGCA TCATTCATTC AAAGCCTTCC AAGCCACGAG ACCCATTTAG TTCCCAAGCC 240
      s59  CTTGCATGCA TCATTCATTC AAAGCCTTCC AAGCCACGAG ACCCATTTAG TTCCCAAGCC 240
    s59-r  CTTGCATGCA TCATTCATTC AAAGCCTTCC AAGCCACGAG ACCCATTTAG TTCCCAAGCC 240
      s51  CTTGCATGCA TCATTCATTC AAAGCCTTCC AAGCCACGAG ACCCATTTAG TTCCCAAGCC 240
    s51-r  CTTGCATGCA TCATTCATTC AAAGCCTTCC AAGCCACGAG ACCCATTTAG TTCCCAAGCC 240
      s36  CTTGCATGCA TCATTCATTC AAAGCCTTCC AAGCCACGAG ACCCATTTAG TTCCCAAGCC 240
    s36-r  CTTGCATGCA TCATTCATTC AAAGCCTTCC AAGCCACGAG ACCCATTTAG TTCCCAAGCC 240
      s33  CTTGCATGCA TCATTCATTC AAAGCCTTCC AAGCCACGAG ACCCATTTAG TTCCCAAGCC 240
    s33-r  CTTGCATGCA TCATTCATTC AAAGCCTTCC AAGCCACGAG ACCCATTTAG TTCCCAAGCC 240
      s26  CTTGCATGCA TCATTCATTC AAAGCCTTCC AAGCCACGAG ACCCATTTAG TTCCCAAGCC 240
    s26-r  CTTGCATGCA TCATTCATTC AAAGCCTTCC AAGCCACGAG ACCCATTTAG TTCCCAAGCC 240
      s19  CTTGCATGCG TCATTCATTC AAAGCCTTCC AAGCCACGAG ACCCATTTAG TTCCCAAGCC 240
    s19-r  CTTGCATGCG TCATTCATTC AAAGCCTTCC AAGCCACGAG ACCCATTTAG TTCCCAAGCC 240
     s110  CTTGCATGCG TCATTCATTC AAAGCCTTCC AAGCCACGAG ACCCATTTAG TTCCCAAGCC 240
   s110-r  CTTGCATGCG TCATTCATTC AAAGCCTTCC AAGCCACGAG ACCCATTTAG TTCCCAAGCC 240
     s170  CTTGCATGCG TCATTCATTC AAAGCCTTCC AAGCCACGAG ACCCATTTAG TTCCCAAGCC 240
   s170-r  CTTGCATGCG TCATTCATTC AAAGCCTTCC AAGCCACGAG ACCCATTTAG TTCCCAAGCC 240
     s109  CTTGCATGCG TCATTCATTC AAAGCCTTCC AAGCCACGAG ACCCATTTAG TTCCCAAGCC 240
   s109-r  CTTGCATGCG TCATTCATTC AAAGCCTTCC AAGCCACGAG ACCCATTTAG TTCCCAAGCC 240
     s168  CTTGCATGCG TCATTCATTC AAAGCCTTCC AAGCCACGAG ACCCATTTAG TTCCCAAGCC 240
   s168-r  CTTGCATGCG TCATTCATTC AAAGCCTTCC AAGCCACGAG ACCCATTTAG TTCCCAAGCC 240
Consensus  CTTGCATGCA TCATTCATTC AAAGCCTTCC AAGCCACGAG ACCCATTTAG TTCCCAAGCC
Conservation (100% / 0%)

                                     260                 280                 300
                                       |                   |                   |
37-contig  TTGTGGTGCT ACTACAACGT CGTCCTACCA AAGCAGTATG AACCCGCAAA GCGTATCCTT 300
      s73  TTGTGGTGCT ACTACAACGT CGTCCTACCA AAGCAGTATG AACCCGCAAA GCGTATCCTT 300
    s73-r  TTGTGGTGCT ACTACAACGT CGTCCTACCA AAGCAGTATG AACCCGCAAA GCGTATCCTT 300
      s81  TTGTGGTGCT ACTACAACGT CGTCCTACCA AAGCAGTATG AACCCGCAAA GCGTATCCTT 300
    s81-r  TTGTGGTGCT ACTACAACGT CGTCCTACCA AAGCAGTATG AACCCGCAAA GCGTATCCTT 300
      s59  TTGTGGTGCT ACTACAACGT CGTCCTACCA AAGCAGTATG AACCCGCAAA GCGTATCCTT 300
    s59-r  TTGTGGTGCT ACTACAACGT CGTCCTACCA AAGCAGTATG AACCCGCAAA GCGTATCCTT 300
      s51  TTGTGGTGCT ACTACAACGT CGTCCTACCA AAGCAGTATG AACCCGCAAA GCGTATCCTT 300
    s51-r  TTGTGGTGCT ACTACAACGT CGTCCTACCA AAGCAGTATG AACCCGCAAA GCGTATCCTT 300
      s36  TTGTGGTGCT ACTACAACGT CGTCCTACCA AAGCAGTATG AACCCGCAAA GCGTATCCTT 300
    s36-r  TTGTGGTGCT ACTACAACGT CGTCCTACCA AAGCAGTATG AACCCGCAAA GCGTATCCTT 300
      s33  TTGTGGTGCT ACTACAACGT CGTCCTACCA AAGCAGTATG AACCCGCAAA GCGTATCCTT 300
    s33-r  TTGTGGTGCT ACTACAACGT CGTCCTACCA AAGCAGTATG AACCCGCAAA GCGTATCCTT 300
      s26  TTGTGGTGCT ACTACAACGT CGTCCTACCA AAGCAGTATG AACCCGCAAA GCGTATCCTT 300
    s26-r  TTGTGGTGCT ACTACAACGT CGTCCTACCA AAGCAGTATG AACCCGCAAA GCGTATCCTT 300
      s19  TTGTGGTGCT ACTACAACGT CGTCCTACCA AAGCAGTATG AACCCGCAAA GCGTATCCTT 300
    s19-r  TTGTGGTGCT ACTACAACGT CGTCCTACCA AAGCAGTATG AACCCGCAAA GCGTATCCTT 300
     s110  TTGTGGTGCT ACTACAACGT CGTCCTACCA AAGCAGTATG AACCCGCAAA GCGTATCCTT 300
   s110-r  TTGTGGTGCT ACTACAACGT CGTCCTACCA AAGCAGTATG AACCCGCAAA GCGTATCCTT 300
     s170  TTGTGGTGCT ACTACAACGT CGTCCTACCA AAGCAGTATG AACCCGCAAA GCGTATCCTT 300
   s170-r  TTGTGGTGCT ACTACAACGT CGTCCTACCA AAGCAGTATG AACCCGCAAA GCGTATCCTT 300
     s109  TTGTGGTGCT ACTACAACGT CGTCCTACCA AAGCAGTATA AACCCGCAAA GCGTATCCTT 300
   s109-r  TTGTGGTGCT ACTACAACGT CGTCCTACCA AAGCAGTATA AACCCGCAAA GCGTATCCTT 300
     s168  TTGTGGTGCT ACTACAACGT CGTCCTACCA AAGCAGTATA AACCCGCAAA GCGTATCCTT 300
   s168-r  TTGTGGTGCT ACTACAACGT CGTCCTACCA AAGCAGTATA AACCCGCAAA GCGTATCCTT 300
Consensus  TTGTGGTGCT ACTACAACGT CGTCCTACCA AAGCAGTATG AACCCGCAAA GCGTATCCTT
Conservation (100% / 0%)

                                     320                 340                 360
                                       |                   |                   |
37-contig  GACACAAAGC TTAAGTAACA AAAATAACAA TTAAAACTCA GACCCTAATA TTCAAATAAT 360
      s73  GACACAAAGC TTAAGTAACA AAAATAACAA TTAAAACTCA GACCCTAATA TTCAAATAAT 360
    s73-r  GACACAAAGC TTAAGTAACA AAAATAACAA TTAAAACTCA GACCCTAATA TTCAAATAAT 360
      s81  GACACAAAGC TTAAGTAACA AAAATAACAA TTAAAACTCA GACCCTAATA TTCAAATAAT 360
    s81-r  GACACAAAGC TTAAGTAACA AAAATAACAA TTAAAACTCA GACCCTAATA TTCAAATAAT 360
      s59  GACACAAAGC TTAAGTAACA AAAATAACAA TTAAAACTCA GACCCTAATA TTCAAATAAT 360
    s59-r  GACACAAAGC TTAAGTAACA AAAATAACAA TTAAAACTCA GACCCTAATA TTCAAATAAT 360
      s51  GACACAAAGC TTAAGTAACA AAAATAACAA TTAAAACTCA GACCCTAATA TTCAAATAAT 360
    s51-r  GACACAAAGC TTAAGTAACA AAAATAACAA TTAAAACTCA GACCCTAATA TTCAAATAAT 360
      s36  GACACAAAGC TTAAGTAACA AAAATAACAA TTAAAACTCA GACCCTAATA TTCAAATAAT 360
    s36-r  GACACAAAGC TTAAGTAACA AAAATAACAA TTAAAACTCA GACCCTAATA TTCAAATAAT 360
      s33  GACACAAAGC TTAAGTAACA AAAATAACAA TTAAAACTCA GACCCTAATA TTCAAATAAT 360
    s33-r  GACACAAAGC TTAAGTAACA AAAATAACAA TTAAAACTCA GACCCTAATA TTCAAATAAT 360
      s26  GACACAAAGC TTAAGTAACA AAAATAACAA TTAAAACTCA GACCCTAATA TTCAAATAAT 360
    s26-r  GACACAAAGC TTAAGTAACA AAAATAACAA TTAAAACTCA GACCCTAATA TTCAAATAAT 360
      s19  GACACAAAGC TTAAGTAACA AAAATAACAA TTAAAACTCA GACCCTAATA TTCAAATAAT 360
    s19-r  GACACAAAGC TTAAGTAACA AAAATAACAA TTAAAACTCA GACCCTAATA TTCAAATAAT 360
     s110  GACACAAAGC TTAAGTAACA AAAATAACAA TTAAAACTCA GACCCTAATA TTCAAATAAT 360
   s110-r  GACACAAAGC TTAAGTAACA AAAATAACAA TTAAAACTCA GACCCTAATA TTCAAATAAT 360
     s170  GACACAAAGC TTAAGTAACA AAAATAACAA TTAAAACTCA GACCCTAATA TTCAAATAAT 360
   s170-r  GACACAAAGC TTAAGTAACA AAAATAACAA TTAAAACTCA GACCCTAATA TTCAAATAAT 360
     s109  GACACAAAGC TTAAGTAACA AAAATAACAA TTAAAATTCA GACCCTAATA TTCAAATAAT 360
   s109-r  GACACAAAGC TTAAGTAACA AAAATAACAA TTAAAATTCA GACCCTAATA TTCAAATAAT 360
     s168  GACACAAAGC TTAAGTAACA AAAATAACAA TTAAAATTCA GACCCTAATA TTCAAATAAT 360
   s168-r  GACACAAAGC TTAAGTAACA AAAATAACAA TTAAAATTCA GACCCTAATA TTCAAATAAT 360
Consensus  GACACAAAGC TTAAGTAACA AAAATAACAA TTAAAACTCA GACCCTAATA TTCAAATAAT
Conservation (100% / 0%)
```

```
                                             380                           400                           420
                                               |                             |                             |
37-contig  AGGAGTCCCC  AACAAGAAAT  AAAAAAGACA  ATAATTGAAG  AGTCTCTAAT  TATAATAAAT  420
      s73  AGGAGTCCCC  AACAAGAAAT  AAAAAAGACA  ATAATTGAAG  AGTCTCTAAT  TATAATAAAT  420
    s73-r  AGGAGTCCCC  AACAAGAAAT  AAAAAAGACA  ATAATTGAAG  AGTCTCTAAT  TATAATAAAT  420
      s81  AGGAGTCCCC  AACAAGAAAT  AAAAAAGACA  ATAATTGAAG  AGTCTCTAAT  TATAATAAAT  420
    s81-r  AGGAGTCCCC  AACAAGAAAT  AAAAAAGACA  ATAATTGAAG  AGTCTCTAAT  TATAATAAAT  420
      s59  AGGAGTCCCC  AACAAGAAAT  AAAAAAGACA  ATAATTGAAG  AGTCTCTAAT  TATAATAAAT  420
    s59-r  AGGAGTCCCC  AACAAGAAAT  AAAAAAGACA  ATAATTGAAG  AGTCTCTAAT  TATAATAAAT  420
      s51  AGGAGTCCCC  AACAAGAAAT  AAAAAAGACA  ATAATTGAAG  AGTCTCTAAT  TATAATAAAT  420
    s51-r  AGGAGTCCCC  AACAAGAAAT  AAAAAAGACA  ATAATTGAAG  AGTCTCTAAT  TATAATAAAT  420
      s36  AGGAGTCCCC  AACAAGAAAT  AAAAAAGACA  ATAATTGAAG  AGTCTCTAAT  TATAATAAAT  420
    s36-r  AGGAGTCCCC  AACAAGAAAT  AAAAAAGACA  ATAATTGAAG  AGTCTCTAAT  TATAATAAAT  420
      s33  AGGAGTCCCC  AACAAGAAAT  AAAAAAGACA  ATAATTGAAG  AGTCTCTAAT  TATAATAAAT  420
    s33-r  AGGAGTCCCC  AACAAGAAAT  AAAAAAGACA  ATAATTGAAG  AGTCTCTAAT  TATAATAAAT  420
      s26  AGGAGTCCCC  AACAAGAAAT  AAAAAAGACA  ATAATTGAAG  AGTCTCTAAT  TATAATAAAT  420
    s26-r  AGGAGTCCCC  AACAAGAAAT  AAAAAAGACA  ATAATTGAAG  AGTCTCTAAT  TATAATAAAT  420
      s19  AGGAGTCCCC  AACAAGAAAT  AAAAAAGACA  ATAATTGAAG  AGTCTCTAAT  TATAATAAAT  420
    s19-r  AGGAGTCCCC  AACAAGAAAT  AAAAAAGACA  ATAATTGAAG  AGTCTCTAAT  TATAATAAAT  420
     s110  AGGAGTCCCC  AACAAGAAAT  AAAAAAGACA  ATAATTGAAG  AGTCTCTAAT  TATAATAAAT  420
   s110-r  AGGAGTCCCC  AACAAGAAAT  AAAAAAGACA  ATAATTGAAG  AGTCTCTAAT  TATAATAAAT  420
     s170  AGGAGTCCCC  AACAAGAAAT  AAAAAAGACA  ATAATTGAAG  AGTCTCTAAT  TATAATAAAT  420
   s170-r  AGGAGTCCCC  AACAAGAAAT  AAAAAAGACA  ATAATTGAAG  AGTCTCTAAT  TATAATAAAT  420
     s109  AGGAGTCCCC  AACAAGAAAT  AAAAAAGACA  ATAATTGAAG  AGTCTCTAAT  TATAATAAAT  420
   s109-r  AGGAGTCCCC  AACAAGAAAT  AAAAAAGACA  ATAATTGAAG  AGTCTCTAAT  TATAATAAAT  420
     s168  AGGAGTCCCC  AACAAGAAAT  AAAAAAGACA  ATAATTGAAG  AGTCTCTAAT  TATAATAAAT  420
   s168-r  AGGAGTCCCC  AACAAGAAAT  AAAAAAGACA  ATAATTGAAG  AGTCTCTAAT  TATAATAAAT  420
Consensus  AGGAGTCCCC  AACAAGAAAT  AAAAAAGACA  ATAATTGAAG  AGTCTCTAAT  TATAATAAAT
```

Conservation

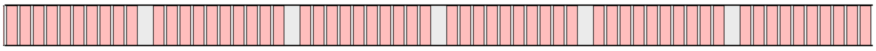


```
                                             440
                                               |
37-contig  GGATGATGTG  ACGAAAAAAT  AAAA  444
      s73  GGATGATGTG  ACGAAAAAAT  AAAA  444
    s73-r  GGATGATGTG  ACGAAAAAAT  AAAA  444
      s81  GGATGATGTG  ACGAAAAAAT  AAAA  444
    s81-r  GGATGATGTG  ACGAAAAAAT  AAAA  444
      s59  GGATGATGTG  ACGAAAAAAT  AAAA  444
    s59-r  GGATGATGTG  ACGAAAAAAT  AAAA  444
      s51  GGATGATGTG  ACGAAAAAAT  AAAA  444
    s51-r  GGATGATGTG  ACGAAAAAAT  AAAA  444
      s36  GGATGATGTG  ACGAAAAAAT  AAAA  444
    s36-r  GGATGATGTG  ACGAAAAAAT  AAAA  444
      s33  GGATGATGTG  ACGAAAAAAT  AAAA  444
    s33-r  GGATGATGTG  ACGAAAAAAT  AAAA  444
      s26  GGATGATGTG  ACGAAAAAAT  AAAA  444
    s26-r  GGATGATGTG  ACGAAAAAAT  AAAA  444
      s19  GGATGATGTG  ACGAAAAAAT  AAAA  444
    s19-r  GGATGATGTG  ACGAAAAAAT  AAAA  444
     s110  GGATGATGTG  ACGAAAAAAT  AAAA  444
   s110-r  GGATGATGTG  ACGAAAAAAT  AAAA  444
     s170  GGATGATGTG  ACGAAAAAAT  AAAA  444
   s170-r  GGATGATGTG  ACGAAAAAAT  AAAA  444
     s109  GGATGATGTG  ACGAAAAAAT  AAAA  444
   s109-r  GGATGATGTG  ACGAAAAAAT  AAAA  444
     s168  GGATGATGTG  ACGAAAAAAT  AAAA  444
   s168-r  GGATGATGTG  ACGAAAAAAT  AAAA  444
Consensus  GGATGATGTG  ACGAAAAAAT  AAAA
```

Conservation

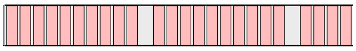

```
                                   20                  40                  60
                                    |                   |                   |
59-contig  ATTGAGACTT AGAGTTTTCA ACGCATGAAT GTAATTATCA AGTGAATATT TTTTGCGTGT 60
     s100  ATTGAGACTT AGAGTTTTCA ACGCATGAAT GTAATTATCA AGTGAATATT TTTTGCGTGT 60
   s100-r  ATTGAGACTT AGAGTTTTCA ACGCATGAAT GTAATTATCA AGTGAATATT TTTTGCGTGT 60
      s86  ATTGAGACTT AGAGTTTTCA ACGCATGAAT GTAATTATCA AGTGAATATT TTTTGCGTGT 60
    s86-r  ATTGAGACTT AGAGTTTTCA ACGCATGAAT GTAATTATCA AGTGAATATT TTTTGCGTGT 60
      s73  ATTGAGACTT AGAGTTTTCA ACGCATGAAT GTAATTATCA AGTGAATATT TTTTGCGTGT 60
    s73-r  ATTGAGACTT AGAGTTTTCA ACGCATGAAT GTAATTATCA AGTGAATATT TTTTGCGTGT 60
     s137  ATTGAGACTT AGAGTTTTCA ACGCATGAAT GTAATTATCA AGTGAATATT TTTTGCGTGT 60
   s137-r  ATTGAGACTT AGAGTTTTCA ACGCATGAAT GTAATTATCA AGTGAATATT TTTTGCGTGT 60
     s173  ATTGAGACTT AGAGTTTTCA ACGCATGAAT GTAATTATCA AGTGAATATT TTTTGCGTGT 60
   s173-r  ATTGAGACTT AGAGTTTTCA ACGCATGAAT GTAATTATCA AGTGAATATT TTTTGCGTGT 60
Consensus  ATTGAGACTT AGAGTTTTCA ACGCATGAAT GTAATTATCA AGTGAATATT TTTTGCGTGT
     100%
Conservation
       0%

                                   80                 100                 120
                                    |                   |                   |
59-contig  ATCTAGAGTA TAATCAAAGA CTAAATTCTT TTGAGACAAA GATTACATTT CTCTCAACAA 120
     s100  ATCTAGAGTA TAATCAAAGA CTAAATTCTT TTGAGACAAA GATTACATTT CTCTCAACAA 120
   s100-r  ATCTAGAGTA TAATCAAAGA CTAAATTCTT TTGAGACAAA GATTACATTT CTCTCAACAA 120
      s86  ATCTAGAGTA TAATCAAAGA CTAAATTCTT TTGAGACAAA GATTACATTT CTCTCAACAA 120
    s86-r  ATCTAGAGTA TAATCAAAGA CTAAATTCTT TTGAGACAAA GATTACATTT CTCTCAACAA 120
      s73  ATCTAGAGTA TAATCAAAGA CTAAATTCTT TTGAGACAAA GATTACATTT CTCTCAACAA 120
    s73-r  ATCTAGAGTA TAATCAAAGA CTAAATTCTT TTGAGACAAA GATTACATTT CTCTCAACAA 120
     s137  ATCTAGAGTA TAATCAAAGA CTAAATTCTT TTGAGACAAA GATTACATTT CTCTCAACAA 120
   s137-r  ATCTAGAGTA TAATCAAAGA CTAAATTCTT TTGAGACAAA GATTACATTT CTCTCAACAA 120
     s173  ATCTAGAGTA TAATCAAAGA CTAAATTCTT TTGAGACAAA GATTACATTT CTCTCAACAA 120
   s173-r  ATCTAGAGTA TAATCAAAGA CTAAATTCTT TTGAGACAAA GATTACATTT CTCTCAACAA 120
Consensus  ATCTAGAGTA TAATCAAAGA CTAAATTCTT TTGAGACAAA GATTACATTT CTCTCAACAA
     100%
Conservation
       0%

                                  140                 160                 180
                                    |                   |                   |
59-contig  AGTTTTTTTA TGTGCATAAT TATATGATA- GGAATAGGAG AGGTACCTCA AAACTACCAG 179
     s100  AGTTTTTTTA TGTGCATAAT TATATGATA- GGAATAGGAG AGGTACCTCA AAACTACCAG 179
   s100-r  AGTTTTTTTA TGTGCATAAT TATATGATA- GGAATAGGAG AGGTACCTCA AAACTACCAG 179
      s86  AGTTTTTTTA TGTGCATAAT TATATGATA- GGAATAGGAG AGGTACCTCA AAACTACCAG 179
    s86-r  AGTTTTTTTA TGTGCATAAT TATATGATA- GGAATAGGAG AGGTACCTCA AAACTACCAG 179
      s73  AGTTTTTTTA TGTGCATAAT TATATGATA- GGAATAGGAG AGGTACCTCA AAACTACCAG 179
    s73-r  AGTTTTTTTA TGTGCATAAT TATATGATA- GGAATAGGAG AGGTACCTCA AAACTACCAG 179
     s137  AGTTTTTTTA TGTGCATAAT TATATGATA- GGAATAGGAG AGGTACCTCA AAACTACCAG 179
   s137-r  AGTTTTTTTA TGTGCATAAT TATATGATA- GGAATAGGAG AGGTACCTCA AAACTACCAG 179
     s173  AGTTTTTTTA TGTGCATAAT TATATGATAG GGAATAGGAG AGGTACCTCA AAACTACCAG 180
   s173-r  AGTTTTTTTA TGTGCATAAT TATATGATAG GGAATAGGAG AGGTACCTCA AAACTACCAG 180
Consensus  AGTTTTTTTA TGTGCATAAT TATATGATA- GGAATAGGAG AGGTACCTCA AAACTACCAG
     100%
Conservation
       0%

                                  200                 220                 240
                                    |                   |                   |
59-contig  AGAGGGCGCC ACTAGCATCC CACTTTTAAC ATAGGCTTAA CCCAGAGGAT TACTGGTCAA 239
     s100  AGAGGGCGCC ACTAGCATCC CACTTTTAAC ATAGGCTTAA CCCAGAGGAT TACTGGTCAA 239
   s100-r  AGAGGGCGCC ACTAGCATCC CACTTTTAAC ATAGGCTTAA CCCAGAGGAT TACTGGTCAA 239
      s86  AGAGGGCGCC ACTAGCATCC CACTTTTAAC ATAGGCTTAA CCCAGAGGAT TACTGGTCAA 239
    s86-r  AGAGGGCGCC ACTAGCATCC CACTTTTAAC ATAGGCTTAA CCCAGAGGAT TACTGGTCAA 239
      s73  AGAGGGCGCC ACTAGCATCC CACTTTTAAC ATAGGCTTAA CCCAGAGGAT TACTGGTCAA 239
    s73-r  AGAGGGCGCC ACTAGCATCC CACTTTTAAC ATAGGCTTAA CCCAGAGGAT TACTGGTCAA 239
     s137  AGAGGGCGCC ACTAGCATCC CACTTTTAAC ATAGGCTTAA CCCAGAGGAT TACTGGTCAA 239
   s137-r  AGAGGGCGCC ACTAGCATCC CACTTTTAAC ATAGGCTTAA CCCAGAGGAT TACTGGTCAA 239
     s173  AGAGGGCGCC ACTAGCATCC CACTTTTAAC ATAGGCTTAA CCCAGAGGAT TACTGGTCAA 240
   s173-r  AGAGGGCGCC ACTAGCATCC CACTTTTAAC ATAGGCTTAA CCCAGAGGAT TACTGGTCAA 240
Consensus  AGAGGGCGCC ACTAGCATCC CACTTTTAAC ATAGGCTTAA CCCAGAGGAT TACTGGTCAA
     100%
Conservation
       0%

59-contig  GCTTGTAT 247
     s100  GCTTGTAT 247
   s100-r  GCTTGTAT 247
      s86  GCTTGTAT 247
    s86-r  GCTTGTAT 247
      s73  GCTTGTAT 247
    s73-r  GCTTGTAT 247
     s137  GCTTGTAT 247
   s137-r  GCTTGTAT 247
     s173  GCTTGTAT 248
   s173-r  GCTTGTAT 248
Consensus  GCTTGTAT
     100%
Conservation
       0%
```

```
                                                  20                  40                  60
                                                  |                   |                   |
88-contig  CTTTTCCTTC ACAATACCGA TGAGTTGTTC CTATGTGGTC AATTCTGTGC TGTCTTTATC  60
s110       CTTTTCCTTC ACAATACCGA TGAGTTGTTC CTATGTGGTC AATTCTGTGC TGTCTTTATC  60
s110-r     CTTTTCCTTC ACAATACCGA TGAGTTGTTC CTATGTGGTC AATTCTGTGC TGTCTTTATC  60
s158       CTTTTCCTTC ACAATACCGA TGAGTTGTTC CTATGTGGTC AATTCTGTGC TGTCTTTATC  60
s158-r     CTTTTCCTTC ACAATACCGA TGAGTTGTTC CTATGTGGTC AATTCTGTGC TGTCTTTATC  60
s86        CTTTTCCTTC ACAATACCGA TGAGTTGTTC CTATGTGGTC AATTCTGTGC TGTCTTTATC  60
s86-r      CTTTTCCTTC ACAATACCGA TGAGTTGTTC CTATGTGGTC AATTCTGTGC TGTCTTTATC  60
s145       CTTTTCCTTC ACAATACCGA TGAGTTGTTC CTATGTGGTC AATTCTGTGC TGTCTTTATC  60
s145-r     CTTTTCCTTC ACAATACCGA TGAGTTGTTC CTATGTGGTC AATTCTGTGC TGTCTTTATC  60
s136       CTTTTCCTTC ACAATACCGA TGAGTTGTTC CTATGTGGTC AATTCTGTGC TGTCTTTATC  60
s136-r     CTTTTCCTTC ACAATACCGA TGAGTTGTTC CTATGTGGTC AATTCTGTGC TGTCTTTATC  60
s172       CTTTTCCTTC ACAATACCGA TGAGTTGTTC CTATGTGGTC AATTCTGTGC TGTCTTTATC  60
s172-r     CTTTTCCTTC ACAATACCGA TGAGTTGTTC CTATGTGGTC AATTCTGTGC TGTCTTTATC  60
s98        CTTTTCCTTC ACAATACCGA TGAGTTGTTC CTATGTGGTC AATTCTGTGC TGTCTTTATC  60
s98-r      CTTTTCCTTC ACAATACCGA TGAGTTGTTC CTATGTGGTC AATTCTGTGC TGTCTTTATC  60
s73        CTTTTCCTTC ACAATACCGA TGAGTTGTTC CTATGTGGTC AATTCTGTGC TGTCTTTATC  60
s73-r      CTTTTCCTTC ACAATACCGA TGAGTTGTTC CTATGTGGTC AATTCTGTGC TGTCTTTATC  60
Consensus  CTTTTCCTTC ACAATACCGA TGAGTTGTTC CTATGTGGTC AATTCTGTGC TGTCTTTATC
Conservation (100% / 0%)

                                                  80                 100                 120
                                                  |                   |                   |
88-contig  AACCCTTGCG TCTTCCACAC CGGCCTTTTT GTCTTGCAGG TACTAATTTT CTCCAAAACA 120
s110       AACCCTTGCG TCTTCCACAC CGGCCTTTTT GTCTTGCAGG TACTAATTTT CTCCAAAACA 120
s110-r     AACCCTTGCG TCTTCCACAC CGGCCTTTTT GTCTTGCAGG TACTAATTTT CTCCAAAACA 120
s158       AACCCTTGCG TCTTCCACAC CGGCCTTTTT GTCTTGCAGG TACTAATTTT CTCCAAAACA 120
s158-r     AACCCTTGCG TCTTCCACAC CGGCCTTTTT GTCTTGCAGG TACTAATTTT CTCCAAAACA 120
s86        AACCCTTGCG TCTTCCACAC CGGCCTTTTT GTCTTGCAGG TACTAATTTT CTCCAAAACA 120
s86-r      AACCCTTGCG TCTTCCACAC CGGCCTTTTT GTCTTGCAGG TACTAATTTT CTCCAAAACA 120
s145       AACCCTTGCG TCTTCCACAC CGGCCTTTTT GTCTTGCAGG TACTAATTTT CTCCAAAACA 120
s145-r     AACCCTTGCG TCTTCCACAC CGGCCTTTTT GTCTTGCAGG TACTAATTTT CTCCAAAACA 120
s136       AACCCTTGCG TCTTCCACAC CGGCCTTTTT GTCTTGCAGG TACTAATTTT CTCCAAAACA 120
s136-r     AACCCTTGCG TCTTCCACAC CGGCCTTTTT GTCTTGCAGG TACTAATTTT CTCCAAAACA 120
s172       AACCCTTGCG TCTTCCACAC CGGCCTTTTT GTCTTGCAGG TACTAATTTT CTCCAAAACA 120
s172-r     AACCCTTGCG TCTTCCACAC CGGCCTTTTT GTCTTGCAGG TACTAATTTT CTCCAAAACA 120
s98        AACCCTTGCG TCTTCCACAC CGGCCTTTTT GTCTTGCAGG TACTAATTTT CTCCAAAACA 120
s98-r      AACCCTTGCG TCTTCCACAC CGGCCTTTTT GTCTTGCAGG TACTAATTTT CTCCAAAACA 120
s73        AACCCTTGCG TCTTCCACAC CGGCCTTTTT GTCTTGCAGG TACTAATTTT CTCCAAAACA 120
s73-r      AACCCTTGCG TCTTCCACAC CGGCCTTTTT GTCTTGCAGG TACTAATTTT CTCCAAAACA 120
Consensus  AACCCTTGCG TCTTCCACAC CGGCCTTTTT GTCTTGCAGG TACTAATTTT CTCCAAAACA
Conservation (100% / 0%)

                                                 140                 160                 180
                                                  |                   |                   |
88-contig  AGTAAACTAC AACAAATCCA ATAACTGCCA AAACTTTAAT ATAACTCCAA ATTTCACTTC 180
s110       AGTAAACTAC AACAAATCCA ATAACTGCCA AAACTTTAAT ATAACTCCAA ATTTCACTTC 180
s110-r     AGTAAACTAC AACAAATCCA ATAACTGCCA AAACTTTAAT ATAACTCCAA ATTTCACTTC 180
s158       AGTAAACTAC AACAAATCCA ATAACTGCCA AAACTTTAAT ATAACTCCAA ATTTCACTTC 180
s158-r     AGTAAACTAC AACAAATCCA ATAACTGCCA AAACTTTAAT ATAACTCCAA ATTTCACTTC 180
s86        AGTAAACTAC AACAAATCCA ATAACTGCCA AAACTTTAAT ATAACTCCAA ATTTCACTTC 180
s86-r      AGTAAACTAC AACAAATCCA ATAACTGCCA AAACTTTAAT ATAACTCCAA ATTTCACTTC 180
s145       AGTAAACTAC AACAAATCCA ATAACTGCCA AAACTTTAAT ATAACTCCAA ATTTCACTTC 180
s145-r     AGTAAACTAC AACAAATCCA ATAACTGCCA AAACTTTAAT ATAACTCCAA ATTTCACTTC 180
s136       AGTAAACTAC AACAAATCCA ATAACTGCCA AAACTTTAAT ATAACTCCAA ATTTCACTTC 180
s136-r     AGTAAACTAC AACAAATCCA ATAACTGCCA AAACTTTAAT ATAACTCCAA ATTTCACTTC 180
s172       AGTAAACTAC AACAAATCCA ATAACTGCCA AAACTTTAAT ATAACTCCAA ATTTCACTTC 180
s172-r     AGTAAACTAC AACAAATCCA ATAACTGCCA AAACTTTAAT ATAACTCCAA ATTTCACTTC 180
s98        AGTAAACTAC AACAAATCCA ATAACTGCCA AAACTTTAAT ATAACTCCAA ATTTCACTTC 180
s98-r      AGTAAACTAC AACAAATCCA ATAACTGCCA AAACTTTAAT ATAACTCCAA ATTTCACTTC 180
s73        AGTAAACTAC AACAAATCCA ATAACTGCCA AAACTTTAAT ATAACTCCAA ATTTCACTTC 180
s73-r      AGTAAACTAC AACAAATCCA ATAACTGCCA AAACTTTAAT ATAACTCCAA ATTTCACTTC 180
Consensus  AGTAAACTAC AACAAATCCA ATAACTGCCA AAACTTTAAT ATAACTCCAA ATTTCACTTC
Conservation (100% / 0%)

                                                 200                 220                 240
                                                  |                   |                   |
88-contig  ATTGGTTATT GCTGCCTTTA AATTGCGTAT TTTAACCAAC AACGGTGTTG GTGTAGTAAT 240
s110       ATTGGTTATT GCTGCCTTTA AATTGCGTAT TTTAACCAAC AACGGTGTTG GTGTAGTAAT 240
s110-r     ATTGGTTATT GCTGCCTTTA AATTGCGTAT TTTAACCAAC AACGGTGTTG GTGTAGTAAT 240
s158       ATTGGTTATT GCTGCCTTTA AATTGCGTAT TTTAACCAAC AACGGTGTTG GTGTAGTAAT 240
s158-r     ATTGGTTATT GCTGCCTTTA AATTGCGTAT TTTAACCAAC AACGGTGTTG GTGTAGTAAT 240
s86        ATTGGTTATT GCTGCCTTTA AATTGCGTAT TTTAACCAAC AACGGTGTTG GTGTAGTAAT 240
s86-r      ATTGGTTATT GCTGCCTTTA AATTGCGTAT TTTAACCAAC AACGGTGTTG GTGTAGTAAT 240
s145       ATTGGTTATT GCTGCCTTTA AATTGCGTAT TTTAACCAAC AACGGTGTTG GTGTAGTAAT 240
s145-r     ATTGGTTATT GCTGCCTTTA AATTGCGTAT TTTAACCAAC AACGGTGTTG GTGTAGTAAT 240
s136       ATTGGTTATT GCTGCCTTTA AATTGCGTAT TTTAACCAAC AACGGTGTTG GTGTAGTAAT 240
s136-r     ATTGGTTATT GCTGCCTTTA AATTGCGTAT TTTAACCAAC AACGGTGTTG GTGTAGTAAT 240
s172       ATTGGTTATT GCTGCCTTTA AATTGCGTAT TTTAACCAAC AACGGTGTTG GTGTAGTAAT 240
s172-r     ATTGGTTATT GCTGCCTTTA AATTGCGTAT TTTAACCAAC AACGGTGTTG GTGTAGTAAT 240
s98        ATTGTTTATT GCTGCCTTTA AATTGCGTAT TTTAACCAAC AACGGTGTTG GTGTAGTAAT 240
s98-r      ATTGTTTATT GCTGCCTTTA AATTGCGTAT TTTAACCAAC AACGGTGTTG GTGTAGTAAT 240
s73        ATTGTTTATT GCTGCCTTTA AATTGCGTAT TTTAACCAAC AACGGTGTTG GTGTAGTAAT 240
s73-r      ATTGTTTATT GCTGCCTTTA AATTGCGTAT TTTAACCAAC AACGGTGTTG GTGTAGTAAT 240
Consensus  ATTGGTTATT GCTGCCTTTA AATTGCGTAT TTTAACCAAC AACGGTGTTG GTGTAGTAAT
Conservation (100% / 0%)
```

88-contig	AGAGG	245
s110	AGAGG	245
s110-r	AGAGG	245
s158	AGAGG	245
s158-r	AGAGG	245
s86	AGAGG	245
s86-r	AGAGG	245
s145	AGAGG	245
s145-r	AGAGG	245
s136	AGAGG	245
s136-r	AGAGG	245
s172	AGAGG	245
s172-r	AGAGG	245
s98	AGAGG	245
s98-r	AGAGG	245
s73	AGAGG	245
s73-r	AGAGG	245
Consensus	AGAGG	

Conservation

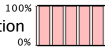

```
                                    20                   40                   60
                                     |                    |                    |
182-contig TGAGGCTAAA AATGGTCGAA TGCGGCAATT AATCTGAGTC CGATGCGGTT TCTGTGATAG 60
      s110 TGAGGCTAAA AATGGTCGAA TGCGGCAATT AATCTGAGTC CGATGCGGTT TCTGTGATAG 60
    s110-r TGAGGCTAAA AATGGTCGAA TGCGGCAATT AATCTGAGTC CGATGCGGTT TCTGTGATAG 60
      s111 TGAGGCTAAA AATGGTCGAA TGCGGCAATT AATCTGAGTC CGATGCGGTT TCTGTGATAG 60
    s111-r TGAGGCTAAA AATGGTCGAA TGCGGCAATT AATCTGAGTC CGATGCGGTT TCTGTGATAG 60
       s86 TGAGGCTAAA AATGGTCGAA TGCGGCAATT AATCTGAGTC CGATGCGGTT TCTGTGATAG 60
     s86-r TGAGGCTAAA AATGGTCGAA TGCGGCAATT AATCTGAGTC CGATGCGGTT TCTGTGATAG 60
      s135 TGAGGCTAAA AATGGTCGAA TGCGGCAATT AATCTGAGTC CGATGCGGTT TCTGTGATAG 60
    s135-r TGAGGCTAAA AATGGTCGAA TGCGGCAATT AATCTGAGTC CGATGCGGTT TCTGTGATAG 60
      s172 TGAGGCTAAA AATGGTCGAA TGCGGCAATT AATCTGAGTC CGATGCGGTT TCTGTGATAG 60
    s172-r TGAGGCTAAA AATGGTCGAA TGCGGCAATT AATCTGAGTC CGATGCGGTT TCTGTGATAG 60
       s73 TGAGGCTAAA AATGGTCGAA TGCGGCAATT AATCTGAGTC CGATGCGGTT TCTGTGATAG 60
     s73-r TGAGGCTAAA AATGGTCGAA TGCGGCAATT AATCTGAGTC CGATGCGGTT TCTGTGATAG 60
      s161 TGAGGCTAAA AATGGTCGAA TGCGGCAATT AATCTGAGTC CGATGCGGTT TCTGTGATAG 60
    s161-r TGAGGCTAAA AATGGTCGAA TGCGGCAATT AATCTGAGTC CGATGCGGTT TCTGTGATAG 60
      s144 TGAGGCTAAA AATGGTCGAA TGCGGCAATT AATCTGAGTC CGATGCGGTT TCTGTGATAG 60
    s144-r TGAGGCTAAA AATGGTCGAA TGCGGCAATT AATCTGAGTC CGATGCGGTT TCTGTGATAG 60
 Consensus TGAGGCTAAA AATGGTCGAA TGCGGCAATT AATCTGAGTC CGATGCGGTT TCTGTGATAG
Conservation (100% / 0%)
```

```
                                    80                  100                  120
                                     |                    |                    |
182-contig TTGAAACTAT GATACTGCGT CGTGATGTTG TGGAGCATAC CTTAACTTGA AACCTTGATA 120
      s110 TTGAAACTAT GATACTGCGT CGTGATGTTG TGGAGCATAC CTTAACTTGA AACCTTGATA 120
    s110-r TTGAAACTAT GATACTGCGT CGTGATGTTG TGGAGCATAC CTTAACTTGA AACCTTGATA 120
      s111 TTGAAACTAT GATACTGCGT CGTGATGTTG TGGAGCATAC CTTAACTTGA AACCTTGATA 120
    s111-r TTGAAACTAT GATACTGCGT CGTGATGTTG TGGAGCATAC CTTAACTTGA AACCTTGATA 120
       s86 TTGAAACTAT GATACTGCGT CGTGATGTTG TGGAGCATAC CTTAACTTGA AACCTTGATA 120
     s86-r TTGAAACTAT GATACTGCGT CGTGATGTTG TGGAGCATAC CTTAACTTGA AACCTTGATA 120
      s135 TTGAAACTAT GATACTGCGT CGTGATGTTG TGGAGCATAC CTTAACTTGA AACCTTGATA 120
    s135-r TTGAAACTAT GATACTGCGT CGTGATGTTG TGGAGCATAC CTTAACTTGA AACCTTGATA 120
      s172 TTGAAACTAT GATACTGCGT CGTGATGCTG TGGAGCATAC CTTAACTTGA AACCTTGATA 120
    s172-r TTGAAACTAT GATACTGCGT CGTGATGCTG TGGAGCATAC CTTAACTTGA AACCTTGATA 120
       s73 TTGAAACTAT GATACTGCGT CGTGATGTTG TGGAGCATAC CTTAACTTGA AACCTTGATA 120
     s73-r TTGAAACTAT GATACTGCGT CGTGATGTTG TGGAGCATAC CTTAACTTGA AACCTTGATA 120
      s161 TTGAAACTAT GATACTGCGT CGTGATGTTG TGGAGCATAC CTTAACTTGA AACCTTGATA 120
    s161-r TTGAAACTAT GATACTGCGT CGTGATGTTG TGGAGCATAC CTTAACTTGA AACCTTGATA 120
      s144 TTGAAACTAT GATACTGCGT CGTGATGTTG TGGAGCATAC CTTAACTTGA AACCTTGATA 120
    s144-r TTGAAACTAT GATACTGCGT CGTGATGTTG TGGAGCATAC CTTAACTTGA AACCTTGATA 120
 Consensus TTGAAACTAT GATACTGCGT CGTGATGTTG TGGAGCATAC CTTAACTTGA AACCTTGATA
Conservation (100% / 0%)
```

```
                                   140                  160                  180
                                     |                    |                    |
182-contig AGTACCAAGA TTTCAAATTA AGCTTCAATC CACGACATCA CGCCACAATA TCATACTTTC 180
      s110 AGTACCAAGA TTTCAAATTA AGCTTCAATC CACGACATCA CGCCACAATA TCATACTTTC 180
    s110-r AGTACCAAGA TTTCAAATTA AGCTTCAATC CACGACATCA CGCCACAATA TCATACTTTC 180
      s111 AGTACCAAGA TTTCAAATTA AGCTTCAATC CACGACATCA CGCCACAATA TCATACTTTC 180
    s111-r AGTACCAAGA TTTCAAATTA AGCTTCAATC CACGACATCA CGCCACAATA TCATACTTTC 180
       s86 AGTACCAAGA TTTCAAATTA AGCTTCAATC CACGACATCA CGCCACAATA TCATACTTTC 180
     s86-r AGTACCAAGA TTTCAAATTA AGCTTCAATC CACGACATCA CGCCACAATA TCATACTTTC 180
      s135 AGTACCAAGA TTTCAAATTA AGCTTCAATC CACGACATCA CGCCACAATA TCATACTTTC 180
    s135-r AGTACCAAGA TTTCAAATTA AGCTTCAATC CACGACATCA CGCCACAATA TCATACTTTC 180
      s172 AGTACCAAGA TTTCAAATTA AGCTTCAATC CACGACATCA CGCCACAATA TCATACTTTC 180
    s172-r AGTACCAAGA TTTCAAATTA AGCTTCAATC CACGACATCA CGCCACAATA TCATACTTTC 180
       s73 AGTACCAAGA TTTCAAATTA AGCTTCAATC CACGACATCA CGCCACAATA TCATACTTTC 180
     s73-r AGTACCAAGA TTTCAAATTA AGCTTCAATC CACGACATCA CGCCACAATA TCATACTTTC 180
      s161 AGTACCAAGA TTTCAAATTA AGCTTCAATC CACGACATCA CGCCACAATA TCATACTTTC 180
    s161-r AGTACCAAGA TTTCAAATTA AGCTTCAATC CACGACATCA CGCCACAATA TCATACTTTC 180
      s144 AGTACCAAGA TTTCAAATTA AGCTTCAATC CACGACATCA CGCCACAATA TCATACTTTC 180
    s144-r AGTACCAAGA TTTCAAATTA AGCTTCAATC CACGACATCA CGCCACAATA TCATACTTTC 180
 Consensus AGTACCAAGA TTTCAAATTA AGCTTCAATC CACGACATCA CGCCACAATA TCATACTTTC
Conservation (100% / 0%)
```

```
                                   200                  220                  240
                                     |                    |                    |
182-contig AGTATTCACA AAAATCGCAT CGGATCATGA TTGTAGACCG CAACAGCCCA TTTGACTGCA 240
      s110 AGTATTCACA AAAATCGCAT CGGATCATGA TTGTAGACCG CAACAGCCCA TTTGACTGCA 240
    s110-r AGTATTCACA AAAATCGCAT CGGATCATGA TTGTAGACCG CAACAGCCCA TTTGACTGCA 240
      s111 AGTATTCACA AAAATCGCAT CGGATCATGA TTGTAGACCG CAACAGCCCA TTTGACTGCA 240
    s111-r AGTATTCACA AAAATCGCAT CGGATCATGA TTGTAGACCG CAACAGCCCA TTTGACTGCA 240
       s86 AGTATTCACA AAAATCGCAT CGGATCATGA TTGTAGACCG CAACAGCCCA TTTGACTGCA 240
     s86-r AGTATTCACA AAAATCGCAT CGGATCATGA TTGTAGACCG CAACAGCCCA TTTGACTGCA 240
      s135 AGTATTCACA AAAATCGCAT CGGATCATGA TTGTAGATCG CAACAGCCCA TTTGACTGCA 240
    s135-r AGTATTCACA AAAATCGCAT CGGATCATGA TTGTAGATCG CAACAGCCCA TTTGACTGCA 240
      s172 AGTATTCACA AAAATCGCAT CGGATCATGA TTGTAGACCG CAACAGCCCA TTTGACTGCA 240
    s172-r AGTATTCACA AAAATCGCAT CGGATCATGA TTGTAGACCG CAACAGCCCA TTTGACTGCA 240
       s73 AGTATTCACA AAAATCGCAT CGGATCATGA TTGTAGACCG CAACAGCCCA TTTGACTGCA 240
     s73-r AGTATTCACA AAAATCGCAT CGGATCATGA TTGTAGACCG CAACAGCCCA TTTGACTGCA 240
      s161 AGTATTCACA AAAATCGCAT CGGATCATGA TTGTAGACCG CAACAGCCCA TTTGACTGCA 240
    s161-r AGTATTCACA AAAATCGCAT CGGATCATGA TTGTAGACCG CAACAGCCCA TTTGACTGCA 240
      s144 AGTATTCACA AAAATCGCAT CGGATCATGA TTGTAGATCG CAACAGCCCA TTTGACTGCA 240
    s144-r AGTATTCACA AAAATCGCAT CGGATCATGA TTGTAGATCG CAACAGCCCA TTTGACTGCA 240
 Consensus AGTATTCACA AAAATCGCAT CGGATCATGA TTGTAGACCG CAACAGCCCA TTTGACTGCA
Conservation (100% / 0%)
```

```
                                          260                                   280                                   300
                                           |                                     |                                     |
182-contig  TTTAACAAAA  GGGACTATAA  CAGCAGTATG  ATCACAACTG  CATTTTGCAA  CCTAGATAAG  300
      s110  TTTAACAAAA  GGGACTATAA  CAGCAGTATG  ATCACAACTG  CATTTTGCAA  CCTAGATAAG  300
    s110-r  TTTAACAAAA  GGGACTATAA  CAGCAGTATG  ATCACAACTG  CATTTTGCAA  CCTAGATAAG  300
      s111  TTTAACAAAA  GGGACTATAA  CAGCAGTATG  ATCACAACTG  CATTTTGCAA  CCTAGATAAG  300
    s111-r  TTTAACAAAA  GGGACTATAA  CAGCAGTATG  ATCACAACTG  CATTTTGCAA  CCTAGATAAG  300
       s86  TTTAACAAAA  GGGACTATAA  CAGCAGTATG  ATCACAACTG  CATTTTGCAA  CCTAGATAAG  300
     s86-r  TTTAACAAAA  GGGACTATAA  CAGCAGTATG  ATCACAACTG  CATTTTGCAA  CCTAGATAAG  300
      s135  TTTAACAAAA  GGGACTATAA  CAGCAGTATG  ATCACAACTG  CATTTTGCAA  CCTAGATAAG  300
    s135-r  TTTAACAAAA  GGGACTATAA  CAGCAGTATG  ATCACAACTG  CATTTTGCAA  CCTAGATAAG  300
      s172  TTTAACAAAA  GGGACTATAA  CAGCAGTATG  ATCACAACTG  CATTTTGCAA  CCTAGATAAG  300
    s172-r  TTTAACAAAA  GGGACTATAA  CAGCAGTATG  ATCACAACTG  CATTTTGCAA  CCTAGATAAG  300
       s73  TTTAACAAAA  GGGACTATAA  CAGCAGTATG  ATCACAACTG  CATTTTGCAA  CCTAGATAAG  300
     s73-r  TTTAACAAAA  GGGACTATAA  CAGCAGTATG  ATCACAACTG  CATTTTGCAA  CCTAGATAAG  300
      s161  TTTAACAAAA  GGGACTATAA  CAGCAGTATG  ATCACAACTG  CATTTTGCAA  CCTAGATAAG  300
    s161-r  TTTAACAAAA  GGGACTATAA  CAGCAGTATG  ATCACAACTG  CATTTTGCAA  CCTAGATAAG  300
      s144  TTTAACAAAA  GGGACTATAA  CAGCAGTATG  ATCACAACTG  CATTTTGCAA  CCTAGATAAG  300
    s144-r  TTTAACAAAA  GGGACTATAA  CAGCAGTATG  ATCACAACTG  CATTTTGCAA  CCTAGATAAG  300

 Consensus  TTTAACAAAA  GGGACTATAA  CAGCAGTATG  ATCACAACTG  CATTTTGCAA  CCTAGATAAG
```

Conservation

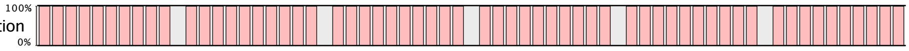

```
                                          320                                   340
                                           |                                     |
182-contig  TACTAACTTG  CAGTTTTGCA  AGATTTACAG  CATATAAACA  TAGACAATAT  CTT  353
      s110  TACTAACTTG  CAGTTTTGCA  AGATTTACAG  CATATAAACA  TAGACAATAT  CTT  353
    s110-r  TACTAACTTG  CAGTTTTGCA  AGATTTACAG  CATATAAACA  TAGACAATAT  CTT  353
      s111  TACTAACTTG  CAGTTTTGCA  AGATTTACAG  CATATAAACA  TAGACAATAT  CTT  353
    s111-r  TACTAACTTG  CAGTTTTGCA  AGATTTACAG  CATATAAACA  TAGACAATAT  CTT  353
       s86  TACTAACTTG  CAGTTTTGCA  AGATTTACAG  CATATAAACA  TAGACAATAT  CTT  353
     s86-r  TACTAACTTG  CAGTTTTGCA  AGATTTACAG  CATATAAACA  TAGACAATAT  CTT  353
      s135  TACTAACTTG  CAGTTTTGCA  AGATTTACAG  CATATAAACA  TAGACAATAT  CTT  353
    s135-r  TACTAACTTG  CAGTTTTGCA  AGATTTACAG  CATATAAACA  TAGACAATAT  CTT  353
      s172  TACTAACTTG  CAGTTTTGCA  AGATTTACAG  CATATAAACA  TAGACAATAT  CTT  353
    s172-r  TACTAACTTG  CAGTTTTGCA  AGATTTACAG  CATATAAACA  TAGACAATAT  CTT  353
       s73  TACTAACTTG  CAGTTTTGCA  AGATTTACAG  CATATAAACA  TAGACAATAT  CTT  353
     s73-r  TACTAACTTG  CAGTTTTGCA  AGATTTACAG  CATATAAACA  TAGACAATAT  CTT  353
      s161  TACTAACTTG  CAGTTTTGCA  AGATTTACAG  CATATAAACA  TAGACAATAT  CTT  353
    s161-r  TACTAACTTG  CAGTTTTGCA  AGATTTACAG  CATATAAACA  TAGACAATAT  CTT  353
      s144  TACTAACTTG  CAGTTTTGCA  AGATTTACAG  CATATAAACA  TAGACAATAT  CTT  353
    s144-r  TACTAACTTG  CAGTTTTGCA  AGATTTACAG  CATATAAACA  TAGACAATAT  CTT  353

 Consensus  TACTAACTTG  CAGTTTTGCA  AGATTTACAG  CATATAAACA  TAGACAATAT  CTT
```

Conservation

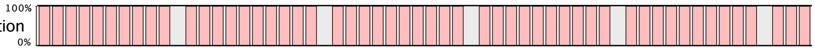

```
                                  20                   40                   60
                                  |                    |                    |
188-contig  ATTATTCTTT TAACAAACAA AAAAGGTTTT TGTCATTCGG CTAAAAATTT GACATTATTT  60
       s36  ATTATTCTTT TAACAAACAA AAAAGGTTTT TGTCATTCGG CTAAAAATTT GACATTATTT  60
     s36-r  ATTATTCTTT TAACAAACAA AAAAGGTTTT TGTCATTCGG CTAAAAATTT GACATTATTT  60
       s23  ATTATTCTTT TAACAAACAA AAAAGGTTTT TGTCATTCGG CTAAAAATTT GACATTATTT  60
     s23-r  ATTATTCTTT TAACAAACAA AAAAGGTTTT TGTCATTCGG CTAAAAATTT GACATTATTT  60
      s189  ATTATTCTTT TAACAAACAA AAAAGGTTTT TGTCATTCGG CTAAAAATTT GACATTATTT  60
    s18p-r  ATTATTCTTT TAACAAACAA AAAAGGTTTT TGTCATTCGG CTAAAAATTT GACATTATTT  60
      s110  ATTATTCTTT TAACAAACAA AAAAGGTTTT TGTCATTCGG CTAAAAATTT GACATTATTT  60
    s110-r  ATTATTCTTT TAACAAACAA AAAAGGTTTT TGTCATTCGG CTAAAAATTT GACATTATTT  60
 Consensus  ATTATTCTTT TAACAAACAA AAAAGGTTTT TGTCATTCGG CTAAAAATTT GACATTATTT
      100%
Conservation
        0%
                                  80                  100                  120
                                  |                    |                    |
188-contig  CAAAAAGTTT CAAAATCACT TTTCACAACT TTAAATTATC TTAAAAATCG TTTTTGACAG  120
       s36  CAAAAAGTTT CAAAATCACT TTTCACAACT TTAAATTATC TTAAAAATCG TTTTTGACAG  120
     s36-r  CAAAAAGTTT CAAAATCACT TTTCACAACT TTAAATTATC TTAAAAATCG TTTTTGACAG  120
       s23  CAAAAAGTTT CAAAATCACT TTTCACAACT TTAAATTATC TTAAAAATCG TTTTTGACAG  120
     s23-r  CAAAAAGTTT CAAAATCACT TTTCACAACT TTAAATTATC TTAAAAATCG TTTTTGACAG  120
      s189  CAAAAAGTTT CAAAATCACT TTTCACAACT TTAAATTATC TTAAAAATCG TTTTTGACAG  120
    s18p-r  CAAAAAGTTT CAAAATCACT TTTCACAACT TTAAATTATC TTAAAAATCG TTTTTGACAG  120
      s110  CAAAAAGTTT CAAAATCACT TTTCACAACT TTAAATTATC TTAAAAATCG TTTTTGACAG  120
    s110-r  CAAAAAGTTT CAAAATCACT TTTCACAACT TTAAATTATC TTAAAAATCG TTTTTGACAG  120
 Consensus  CAAAAAGTTT CAAAATCACT TTTCACAACT TTAAATTATC TTAAAAATCG TTTTTGACAG
      100%
Conservation
        0%
                                 140                  160                  180
                                  |                    |                    |
188-contig  CAAATATATC CAAACCAAAA TTAAATAAGT ATTAGACAAT CAATTTTTTT CATAGCTTAT  180
       s36  CAAATATATC CAAACCAAAA TTAAATAAGT ATTAGACAAT CAATTTTTTT CATAGCTTAT  180
     s36-r  CAAATATATC CAAACCAAAA TTAAATAAGT ATTAGACAAT CAATTTTTTT CATAGCTTAT  180
       s23  CAAATATATC CAAACCAAAA TTAAATAAGT ATTAGACAAT CAATTTTTTT CATAGCTTAT  180
     s23-r  CAAATATATC CAAACCAAAA TTAAATAAGT ATTAGACAAT CAATTTTTTT CATAGCTTAT  180
      s189  CAAATATATC CAAACCAAAA TTAAATAAGT ATTAGACAAT CAATTTTTTT CATAGCTTAT  180
    s18p-r  CAAATATATC CAAACCAAAA TTAAATAAGT ATTAGACAAT CAATTTTTTT CATAGCTTAT  180
      s110  CAAATATATC CAAACCAAAA TTAAATAAGT ATTAGACAAT CAATTTTTTT CATAGCTTAT  180
    s110-r  CAAATATATC CAAACCAAAA TTAAATAAGT ATTAGACAAT CAATTTTTTT CATAGCTTAT  180
 Consensus  CAAATATATC CAAACCAAAA TTAAATAAGT ATTAGACAAT CAATTTTTTT CATAGCTTAT
      100%
Conservation
        0%
                                 200                  220                  240
                                  |                    |                    |
188-contig  TTTCACAATT AATTTTACAC ACTCTAAAAA AACTTTTTTA AAAGCTTAAA TTTGACTTAT  240
       s36  TTTCACAATT AATTTTACAC ACTCTAAAAA AACTTTTTTA AAAGCTTAAA TTTGACTTAT  240
     s36-r  TTTCACAATT AATTTTACAC ACTCTAAAAA AACTTTTTTA AAAGCTTAAA TTTGACTTAT  240
       s23  TTTCACAATT AATTTTACAC ACTCTAAAAA AACTTTTTTA AAAGCTTAAA TTTGACTTAT  240
     s23-r  TTTCACAATT AATTTTACAC ACTCTAAAAA AACTTTTTTA AAAGCTTAAA TTTGACTTAT  240
      s189  TTTCACAATT AATTTTACAC ACTCTAAAAA AACTTTTTTA AAAGCTTAAA TTTGACTTAT  240
    s18p-r  TTTCACAATT AATTTTACAC ACTCTAAAAA AACTTTTTTA AAAGCTTAAA TTTGACTTAT  240
      s110  TTTCACAATT AATTTTACAC ACTCTAAAAA AACTTTTTTA AAAGCTTAAA TTTGACTTAT  240
    s110-r  TTTCACAATT AATTTTACAC ACTCTAAAAA AACTTTTTTA AAAGCTTAAA TTTGACTTAT  240
 Consensus  TTTCACAATT AATTTTACAC ACTCTAAAAA AACTTTTTTA AAAGCTTAAA TTTGACTTAT
      100%
Conservation
        0%
                                 260                  280                  300
                                  |                    |                    |
188-contig  TAATATAAAA ACCAGACTAA ACTTTAAATA GGTCAAGCCA CAGATAGTTG ACAACCACCT  300
       s36  TAATATAAAG ACCAGACTAA ACTTTAAATA GGTCAAGCCA CAGACAGTTG ACAACCACCT  300
     s36-r  TAATATAAAG ACCAGACTAA ACTTTAAATA GGTCAAGCCA CAGACAGTTG ACAACCACCT  300
       s23  TAATATAAAA ACCAGACTAA ACTTTAAATA GGTCAAGCCA CAGATAGTTG ACAACCACCT  300
     s23-r  TAATATAAAA ACCAGACTAA ACTTTAAATA GGTCAAGCCA CAGATAGTTG ACAACCACCT  300
      s189  TAATATAAAA ACCAGACTAA ACTTTAAATA GGTCAAGCCA CAGATAGTTG ACAACCACCT  300
    s18p-r  TAATATAAAA ACCAGACTAA ACTTTAAATA GGTCAAGCCA CAGATAGTTG ACAACCACCT  300
      s110  TAATATAAAG ACCAGACTAA ACTTTAAATA GGTCAAGCCA CAGACAGTTG ACAACCACCT  300
    s110-r  TAATATAAAG ACCAGACTAA ACTTTAAATA GGTCAAGCCA CAGACAGTTG ACAACCACCT  300
 Consensus  TAATATAAAA ACCAGACTAA ACTTTAAATA GGTCAAGCCA CAGATAGTTG ACAACCACCT
      100%
Conservation
        0%
                                 320                  340                  360
                                  |                    |                    |
188-contig  AACCTATTCC CACCCGAATT TCGAGTCACA AGCAAAATGA AATAGGGTGT ATATGAGCCA  360
       s36  AACCTATTCC CACCCGTATT TCGAGTCACA AGCAAAATGA AATAGGGTGT ATATGAGCCA  360
     s36-r  AACCTATTCC CACCCGTATT TCGAGTCACA AGCAAAATGA AATAGGGTGT ATATGAGCCA  360
       s23  AACCTATTCC CACCCGAATT TCGAGTCACA AGCAAAATGA AATAGGGTGT ATATGAGCCA  360
     s23-r  AACCTATTCC CACCCGAATT TCGAGTCACA AGCAAAATGA AATAGGGTGT ATATGAGCCA  360
      s189  AACCTATTCC CACCCGAATT TCGAGTCACA AGCAAAATGA AATAGGGTGT ATATGAGCCA  360
    s18p-r  AACCTATTCC CACCCGAATT TCGAGTCACA AGCAAAATGA AATAGGGTGT ATATGAGCCA  360
      s110  AACCTATTCC CACCCGTATT TCGAGTCACA AGCAAAATGA AATAGGGTGT ATATGAGCCA  360
    s110-r  AACCTATTCC CACCCGTATT TCGAGTCACA AGCAAAATGA AATAGGGTGT ATATGAGCCA  360
 Consensus  AACCTATTCC CACCCGAATT TCGAGTCACA AGCAAAATGA AATAGGGTGT ATATGAGCCA
      100%
Conservation
        0%
```

```
                                      380                           400
                                        |                             |
188-contig CATCCTCCAT GACCTAACAT TCGAGATTCT TAATTCTAAT ATGTGTTGTA GAATTTTGA 419
       s36 CATCCTCCAT GACCTAACAT TCGAGATTCT TAATTCTAAT ATGTGTTGTA GAATTTTGA 419
     s36-r CATCCTCCAT GACCTAACAT TCGAGATTCT TAATTCTAAT ATGTGTTGTA GAATTTTGA 419
       s23 CATCCTCCAT GACCTAACAT TCGAGATTCT TAATTCTAAT ATGTGTTGTA GAATTTTGA 419
     s23-r CATCCTCCAT GACCTAACAT TCGAGATTCT TAATTCTAAT ATGTGTTGTA GAATTTTGA 419
      s189 CATCCTCCAT GACCTAACAT TCGAGATTCT TAATTCTAAT ATGTGTTGTA GAATTTTGA 419
    s18p-r CATCCTCCAT GACCTAACAT TCGAGATTCT TAATTCTAAT ATGTGTTGTA GAATTTTGA 419
      s110 CATCCTCCAT GACCTAACAT TCGAGATTCT TAATTCTAAT ATGTGTTGTA GAATTTTGA 419
    s110-r CATCCTCCAT GACCTAACAT TCGAGATTCT TAATTCTAAT ATGTGTTGTA GAATTTTGA 419

 Consensus CATCCTCCAT GACCTAACAT TCGAGATTCT TAATTCTAAT ATGTGTTGTA GAATTTTGA
```

Conservation

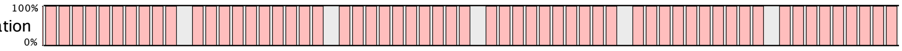

```
                                  20                  40                  60
                                   |                   |                   |
  229-contig TTTTCTTAGT AGTTTGTTAA TAAGATAGCT AAGCATATAT ACTACTTGTA TTCACTTTCT 60
        s110 TTTTCTTAGT AGTTTGTTAA TAAGATAGCT AAGCATATAT ACTACTTGTA TTCACTTTCT 60
      s110-r TTTTCTTAGT AGTTTGTTAA TAAGATAGCT AAGCATATAT ACTACTTGTA TTCACTTTCT 60
        s100 TTTTCTTAGT AGTTTGTTAA TAAGATAGCT AAGCATATAT ACTACTTGTA TTCACTTTCT 60
      s100-r TTTTCTTAGT AGTTTGTTAA TAAGATAGCT AAGCATATAT ACTACTTGTA TTCACTTTCT 60
         s87 TTTTCTTAGT AGTTTGTTAA TAAGATAGCT AAGCATATAT ACTACTTGTA TTCACTTTCT 60
       s87-r TTTTCTTAGT AGTTTGTTAA TAAGATAGCT AAGCATATAT ACTACTTGTA TTCACTTTCT 60
         s73 TTTTCTTAGT AGTTTGTTAA TAAGATAGCT AAGCATATAT ACTACTTGTA TTCACTTTCT 60
       s73-r TTTTCTTAGT AGTTTGTTAA TAAGATAGCT AAGCATATAT ACTACTTGTA TTCACTTTCT 60
        s137 TTTTCTTAGT AGTTTGTTAA TAAGATAGCT AAGCATATAT ACTACTTGTA TTCACTTTCT 60
      s137-r TTTTCTTAGT AGTTTGTTAA TAAGATAGCT AAGCATATAT ACTACTTGTA TTCACTTTCT 60
        s144 TTTTCTTAGT AGTTTGTTAA TAAGATAGCT AAGCATATAT ACTACTTGTA TTCACTTTCT 60
      s144-r TTTTCTTAGT AGTTTGTTAA TAAGATAGCT AAGCATATAT ACTACTTGTA TTCACTTTCT 60
        s172 TTTTCTTAGT AGTTTGTTAA TAAGATAGCT AAGCATATAT ACTACTTGTA TTCACTTTCT 60
      s172-r TTTTCTTAGT AGTTTGTTAA TAAGATAGCT AAGCATATAT ACTACTTGTA TTCACTTTCT 60
        s155 TTTTCTTAGT AGTTTGTTAA TAAGATAGCT AAGCATATAT ACTACTTGTA TTCACTTTCT 60
      s155-r TTTTCTTAGT AGTTTGTTAA TAAGATAGCT AAGCATATAT ACTACTTGTA TTCACTTTCT 60
   Consensus TTTTCTTAGT AGTTTGTTAA TAAGATAGCT AAGCATATAT ACTACTTGTA TTCACTTTCT
        100%
Conservation
          0%
```

```
                                  80                 100                 120
                                   |                   |                   |
  229-contig GCAATTTAAT GAGAACACAC AAATTACAAT ACATCTTTTT CTTCTTCTCT GCGTGTGATC 120
        s110 GCAATTTAAT GAGAACACAC AAATTACAAT ACATCTTTTT CTTCTTCTCT GCGTGTGATC 120
      s110-r GCAATTTAAT GAGAACACAC AAATTACAAT ACATCTTTTT CTTCTTCTCT GCGTGTGATC 120
        s100 GCAATTTAAT GAGAACACAC AAATTACAAT ACATCTTTTT CTTCTTCTCT GCGTGTGATC 120
      s100-r GCAATTTAAT GAGAACACAC AAATTACAAT ACATCTTTTT CTTCTTCTCT GCGTGTGATC 120
         s87 GCAATTTAAT GAGAACACAC AAATTACAAT ACATCTTTTT CTTCTTCTCT GCGTGTGATC 120
       s87-r GCAATTTAAT GAGAACACAC AAATTACAAT ACATCTTTTT CTTCTTCTCT GCGTGTGATC 120
         s73 GCAATTTAAT GAGAACACAC AAATTACAAT ACATCTTTTT CTTCTTCTCT GCGTGTGATC 120
       s73-r GCAATTTAAT GAGAACACAC AAATTACAAT ACATCTTTTT CTTCTTCTCT GCGTGTGATC 120
        s137 GCAATTTAAT GAGAACACAC AAATTACAAT ACATCTTTTT CTTCTTCTCT GCGTGTGATC 120
      s137-r GCAATTTAAT GAGAACACAC AAATTACAAT ACATCTTTTT CTTCTTCTCT GCGTGTGATC 120
        s144 GCAATTTAAT GAGAACACAC AAATTACAAT ACATCTTTTT CTTCTTCTCT GCGTGTGATC 120
      s144-r GCAATTTAAT GAGAACACAC AAATTACAAT ACATCTTTTT CTTCTTCTCT GCGTGTGATC 120
        s172 GCAATTTAAT GAGAACACAC AAATTACAAT ACATCTTTTT CTTCTTCTCT GCGTGTGATC 120
      s172-r GCAATTTAAT GAGAACACAC AAATTACAAT ACATCTTTTT CTTCTTCTCT GCGTGTGATC 120
        s155 GCAATTTAAT GAGAACACAC AAATTACAAT ACATCTTTTT CTTCTTCTCT GCGTGTGATC 120
      s155-r GCAATTTAAT GAGAACACAC AAATTACAAT ACATCTTTTT CTTCTTCTCT GCGTGTGATC 120
   Consensus GCAATTTAAT GAGAACACAC AAATTACAAT ACATCTTTTT CTTCTTCTCT GCGTGTGATC
        100%
Conservation
          0%
```

```
                                 140                 160                 180
                                   |                   |                   |
  229-contig ACTCTGAGTC AAAATGCCTT GCATCCTAAT ATGCAATGGT GTTCTTCATT CAAACATTTA 180
        s110 ACTCTGAGTC AAAATGCCTT GCATCCTAAT ATGCAATGGT GTTCTTCATT CAAACATTTA 180
      s110-r ACTCTGAGTC AAAATGCCTT GCATCCTAAT ATGCAATGGT GTTCTTCATT CAAACATTTA 180
        s100 ACTCTGAGTC AAAATGCCTT GCATCCTAAT ATGCAATGGT GTTCTTCATT CAAACATTTA 180
      s100-r ACTCTGAGTC AAAATGCCTT GCATCCTAAT ATGCAATGGT GTTCTTCATT CAAACATTTA 180
         s87 ACTCTGAGTC AAAATGCCTT GCATCCTAAT ATGCAATGGT GTTCTTCATT CAAACATTTA 180
       s87-r ACTCTGAGTC AAAATGCCTT GCATCCTAAT ATGCAATGGT GTTCTTCATT CAAACATTTA 180
         s73 ACTCTGAGTC AAAATGCCTT GCATCCTAAT ATGCAATGGT GTTCTTCATT CAAACATTTA 180
       s73-r ACTCTGAGTC AAAATGCCTT GCATCCTAAT ATGCAATGGT GTTCTTCATT CAAACATTTA 180
        s137 ACTCTGAGTC AAAATGCCTT GCATCCTAAT ATGCAATGGT GTTCTTCATT CAAACATTTA 180
      s137-r ACTCTGAGTC AAAATGCCTT GCATCCTAAT ATGCAATGGT GTTCTTCATT CAAACATTTA 180
        s144 ACTCTGAGTC AAAATGCCTT GCATCCTAAT ATGCAATGGT GTTCTTCATT CAAACATTTA 180
      s144-r ACTCTGAGTC AAAATGCCTT GCATCCTAAT ATGCAATGGT GTTCTTCATT CAAACATTTA 180
        s172 ACTCTGAGTC AAAATGCCTT GCATCCTAAT ATGCAATGGT GTTCTTCATT CAAACATTTA 180
      s172-r ACTCTGAGTC AAAATGCCTT GCATCCTAAT ATGCAATGGT GTTCTTCATT CAAACATTTA 180
        s155 ACTCTGAGTC AAAATGCCTT GCATCCTAAT ATGCAATGGT GTTCTTCATT CAAACATTTA 180
      s155-r ACTCTGAGTC AAAATGCCTT GCATCCTAAT ATGCAATGGT GTTCTTCATT CAAACATTTA 180
   Consensus ACTCTGAGTC AAAATGCCTT GCATCCTAAT ATGCAATGGT GTTCTTCATT CAAACATTTA
        100%
Conservation
          0%
```

```
                                 200                 220                 240
                                   |                   |                   |
  229-contig CTTGTAATAT GGTATCAGAG TTGAAAGGGC TACAATCATG CCGCAATTCA CGAAGACTCG 240
        s110 CTTGTAATAT GGTATCAGAG TTGAAAGGGC TACAATCATG CCGCAATTCA CGAAGACTCG 240
      s110-r CTTGTAATAT GGTATCAGAG TTGAAAGGGC TACAATCATG CCGCAATTCA CGAAGACTCG 240
        s100 CTTGTAATAT GGTATCAGAG TTGAAAGGGC TACAATCATG CCGCAATTCA CGAAGACTCG 240
      s100-r CTTGTAATAT GGTATCAGAG TTGAAAGGGC TACAATCATG CCGCAATTCA CGAAGACTCG 240
         s87 CTTGTAATAT GGTATCAGAG TTGAAAGGGC TACAATCATG CCGCAATTCA CGAAGACTCG 240
       s87-r CTTGTAATAT GGTATCAGAG TTGAAAGGGC TACAATCATG CCGCAATTCA CGAAGACTCG 240
         s73 CTTGTAATAT GGTATCAGAG TTGAAAGGGC TACAATCATG CCGCAATTCA CGAAGACTCG 240
       s73-r CTTGTAATAT GGTATCAGAG TTGAAAGGGC TACAATCATG CCGCAATTCA CGAAGACTCG 240
        s137 CTTGTAATAT GGTATCAGAG TTGAAAGGGC TACAATCATG CCGCAATTCA CGAAGACTCG 240
      s137-r CTTGTAATAT GGTATCAGAG TTGAAAGGGC TACAATCATG CCGCAATTCA CGAAGACTCG 240
        s144 CTTGTAATAT GGTATCAGAG TTGAAAGGGC TACAATCATG CCGCAATTCA CGAAGACTCG 240
      s144-r CTTGTAATAT GGTATCAGAG TTGAAAGGGC TACAATCATG CCGCAATTCA CGAAGACTCG 240
        s172 CTTGTAATAT GGTATCAGAG TTGAAAGGGC TACAATCATG CCGCAATTCA CGAAGACTCG 240
      s172-r CTTGTAATAT GGTATCAGAG TTGAAAGGGC TACAATCATG CCGCAATTCA CGAAGACTCG 240
        s155 CTTGTAATAT GGTATCAGAG TTGAAAGGGC TACAATCATG CCGCAATTCA CGAAGACTCG 240
      s155-r CTTGTAATAT GGTATCAGAG TTGAAAGGGC TACAATCATG CCGCAATTCA CGAAGACTCG 240
   Consensus CTTGTAATAT GGTATCAGAG TTGAAAGGGC TACAATCATG CCGCAATTCA CGAAGACTCG
        100%
Conservation
          0%
```

```
                                              260                           280                           300
                                               |                             |                             |
229-contig  ATGGCGATAA GTTTGATTTT GGTATATTAT TTTCATTCTA TACTCTGATA CAGAGCGTTT 300
      s110  ATGGCGATAA GTTTGATTTT GGTATATTAT TTTCATTCTA TACTCTGATA CAGAGCGTTT 300
    s110-r  ATGGCGATAA GTTTGATTTT GGTATATTAT TTTCATTCTA TACTCTGATA CAGAGCGTTT 300
      s100  ATGGCGATAA GTTTGATTTT GGTATATTAT TTTCATTCTA TACTCTGATA CAGAGCGTTT 300
    s100-r  ATGGCGATAA GTTTGATTTT GGTATATTAT TTTCATTCTA TACTCTGATA CAGAGCGTTT 300
       s87  ATGGCGATAA GTTTGATTTT GGTATATTAT TTTCATTCTA TACTCTGATA CAGAGCGTTT 300
     s87-r  ATGGCGATAA GTTTGATTTT GGTATATTAT TTTCATTCTA TACTCTGATA CAGAGCGTTT 300
       s73  ATGGCGATAA GTTTGATTTT GGTATATTAT TTTCATTCTA TACTCTGATA CAGAGCGTTT 300
     s73-r  ATGGCGATAA GTTTGATTTT GGTATATTAT TTTCATTCTA TACTCTGATA CAGAGCGTTT 300
      s137  ATGGCGATAA GTTTGATTTT GGTATATTAT TTTCATTCTA TACTCTGATA CAGAGCGTTT 300
    s137-r  ATGGCGATAA GTTTGATTTT GGTATATTAT TTTCATTCTA TACTCTGATA CAGAGCGTTT 300
      s144  ATGGCGATAA GTTTGATTTT GGTATATTAT TTTCATTCTA TACTCTGATA CAGAGCGTTT 300
    s144-r  ATGGCGATAA GTTTGATTTT GGTATATTAT TTTCATTCTA TACTCTGATA CAGAGCGTTT 300
      s172  ATGGCGATAA GTTTGATTTT GGTATATTAT TCTCATTCTA TACTCTGATA CAGAGCGTTT 300
    s172-r  ATGGCGATAA GTTTGATTTT GGTATATTAT TCTCATTCTA TACTCTGATA CAGAGCGTTT 300
      s155  ATGGCGATAA GTTTGATTTT GGTATATTAT TTTCATTCTA TACTCTGATA CAGAGCGTTT 300
    s155-r  ATGGCGATAA GTTTGATTTT GGTATATTAT TTTCATTCTA TACTCTGATA CAGAGCGTTT 300
 Consensus  ATGGCGATAA GTTTGATTTT GGTATATTAT TTTCATTCTA TACTCTGATA CAGAGCGTTT
      100%
Conservation
        0%

                                              320
                                               |
229-contig  GGAATACCTA CTAACATAAG CTTCAGAATA CTTACAGG 338
      s110  GGAATACCTA CTAACATAAG CTTCAGAATA CTTACAGG 338
    s110-r  GGAATACCTA CTAACATAAG CTTCAGAATA CTTACAGG 338
      s100  GGAATACCTA CTAACATAAG CTTCAGAATA CTTACAGG 338
    s100-r  GGAATACCTA CTAACATAAG CTTCAGAATA CTTACAGG 338
       s87  GGAATACCTA CTAACATAAG CTTCAGAATA CTTACAGG 338
     s87-r  GGAATACCTA CTAACATAAG CTTCAGAATA CTTACAGG 338
       s73  GGAATACCTA CTAACATAAG CTTCAGAATA CTTACAGG 338
     s73-r  GGAATACCTA CTAACATAAG CTTCAGAATA CTTACAGG 338
      s137  GGAATACCTA CTAACATAAG CTTCAGAATA CTTACAGG 338
    s137-r  GGAATACCTA CTAACATAAG CTTCAGAATA CTTACAGG 338
      s144  GGAATACCTA CTAACATAAG CTTCAGAATA CTTACAGG 338
    s144-r  GGAATACCTA CTAACATAAG CTTCAGAATA CTTACAGG 338
      s172  GGAATACCTA CTAACATAAG CTTCAGAATA CTTACAGG 338
    s172-r  GGAATACCTA CTAACATAAG CTTCAGAATA CTTACAGG 338
      s155  GGAATACCTA CTAACATAAG CTTCAGAATA CTTACAGG 338
    s155-r  GGAATACCTA CTAACATAAG CTTCAGAATA CTTACAGG 338
 Consensus  GGAATACCTA CTAACATAAG CTTCAGAATA CTTACAGG
      100%
Conservation
        0%
```
