# Supplementary material for: Development of SNP Set for the Marker-Assisted Selection of Guar (Cyamopsis tetragonoloba (L.) Taub.) Based on a Custom Reference Genome Assembly
Source: Plants (Basel). 2021 Sep 30;10(10):2063. doi: 10.3390/plants10102063 (PMC8539970; doi:10.3390/plants10102063)
Supplement: Supplementary file 1 [file plants-10-02063-s001.zip › List of Supplementary Materials.pdf]

Supplementary Materials Table S1.
List of 166 guar plants genotyped by SNP and InDel markers developed in the study.

Supplementary Materials Table S2
Set of 5,234 variants (SNP and InDel) polymorphic between 166 guar plants

Supplementary Materials Figure S1. Construction of a reference guar genome variant dataset.
(a) Summary statistics of the variant sets detected by each of the three tools used (GATK-HC, NGSEP, TASSEL5). From left to right: number of biallelic variants, distribution of non-reference allele frequency (AF), numbers of heterozygous genotypes per site, allele balance for heterozygous genotypes, and tread depth at variant sites. (b). Pairwise concordance between variant datasets. Color represents the overlap index between sets of variants (see Methods). (c) A Venn diagram representing the numbers of variant calls shared by different combinations of variant discovery pipelines. For (b) and (c), the number of shared variant sites was computed as the number of shared variant positions.

Supplementary Materials Figure S2.
The relationship between the non-reference allele frequency and the number of heterozygous genotypes compared to the Hardy-Weinberg equilibrium

Supplementary Materials Figure S3.
Pairwise linkage disequilibrium (r2) values for pairs of SNPs in the guar genotype dataset located at an indicated physical distance in the same contig of the custom guar reference genome assembly.

Supplementary Materials Figure S4.
A heatmap representation of the pairwise Pearson correlation scores between INT-transformed agrobiological traits assessed for guar plants.

Supplementary Materials Figure S5.
Results of GLM-based genome-wide association analysis.

Supplementary Materials Figure S6.
Validation of SNPs and InDel associated with early maturation by PCR and Sanger sequencing.
