# Supplementary figures and images for: Development of SNP Set for the Marker-Assisted Selection of Guar (Cyamopsis tetragonoloba (L.) Taub.) Based on a Custom Reference Genome Assembly
Source: Plants (Basel). 2021 Sep 30;10(10):2063. doi: 10.3390/plants10102063 (PMC8539970; doi:10.3390/plants10102063)

**a**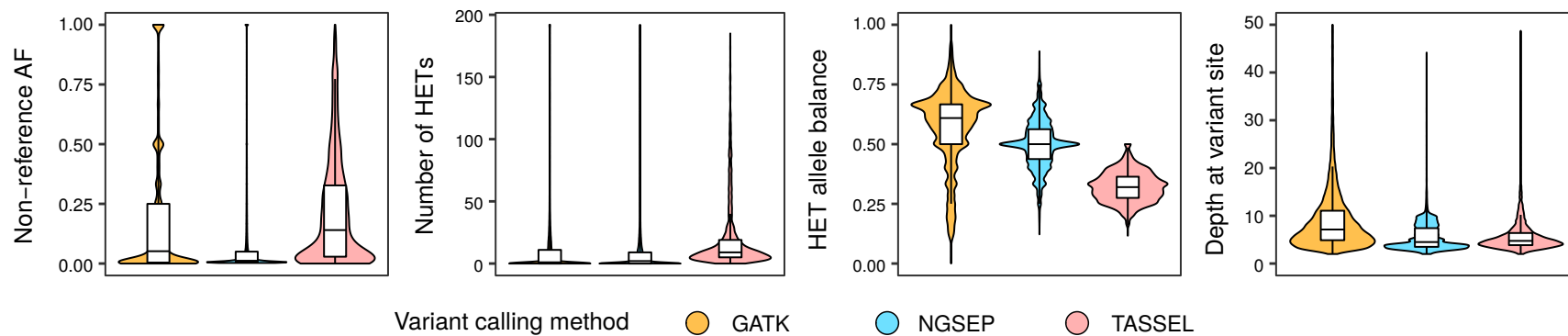**b**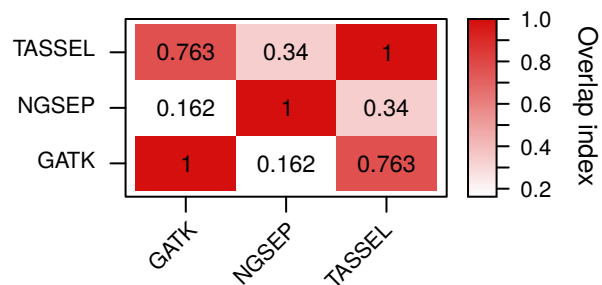**c**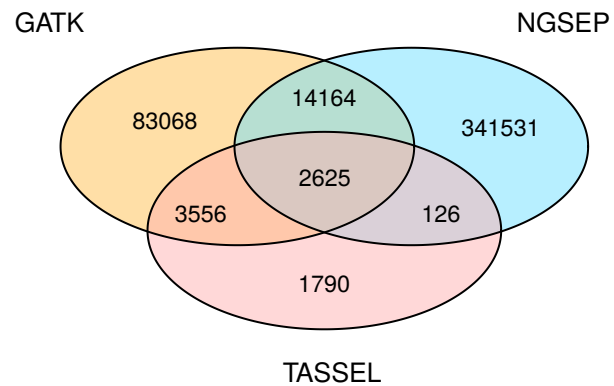

Supplement: Supplementary file 1 [file plants-10-02063-s001.zip › figures/Supplementary Materials Figure S1.pdf]

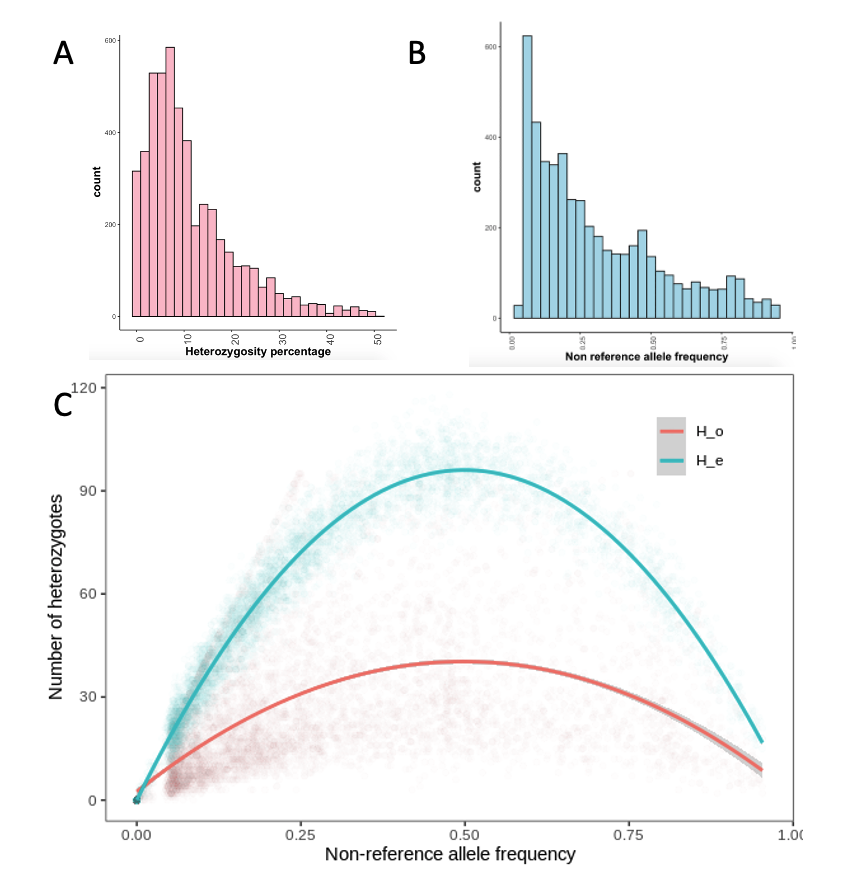

Supplement: Supplementary file 1 [file plants-10-02063-s001.zip › figures/Supplementary Materials Figure S2.png]

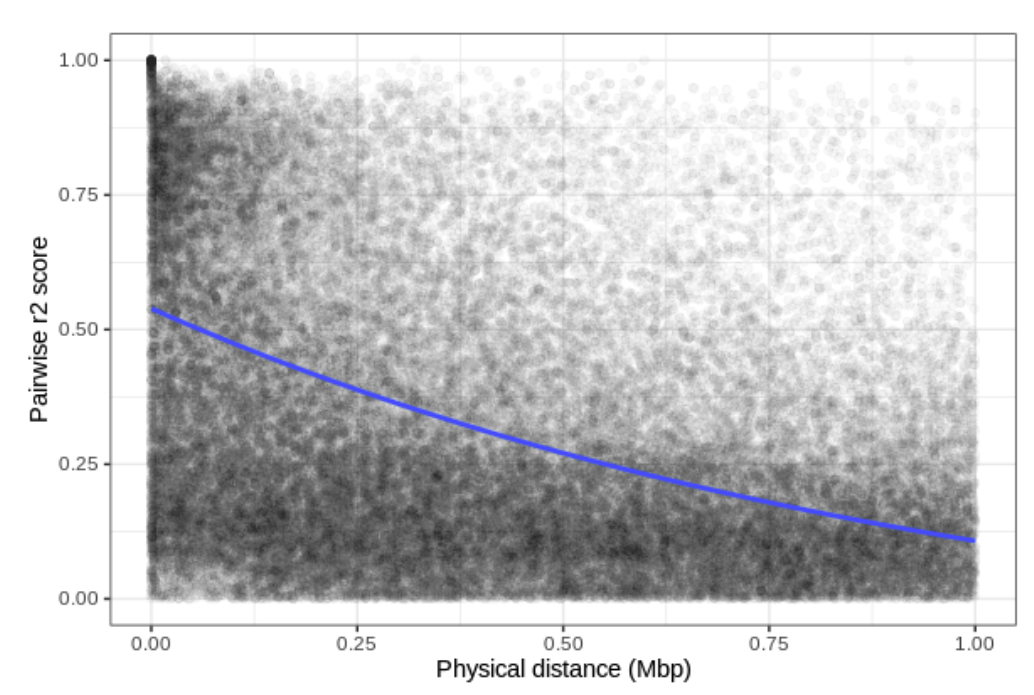

Supplement: Supplementary file 1 [file plants-10-02063-s001.zip › figures/Supplementary Materials Figure S3.png]

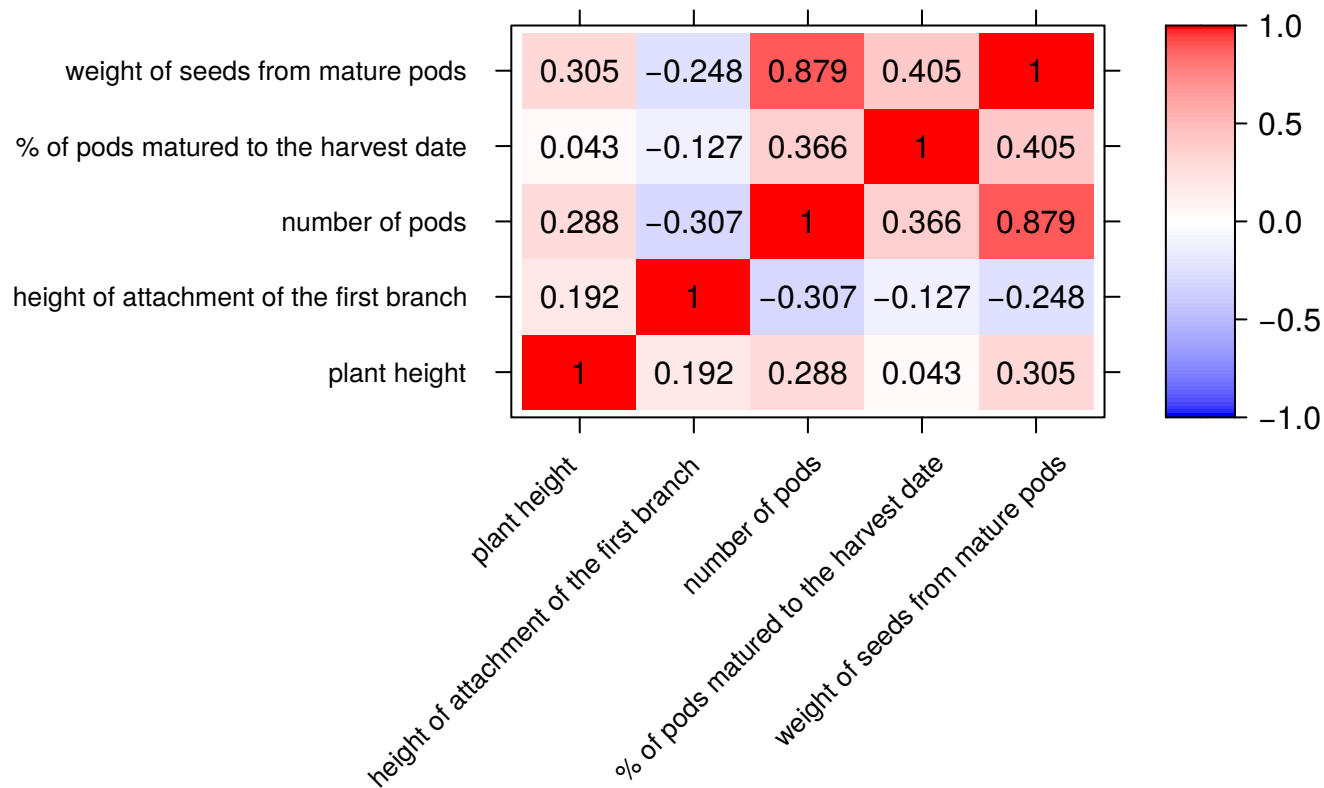

Supplement: Supplementary file 1 [file plants-10-02063-s001.zip › figures/Supplementary Materials Figure S4.pdf]

**a**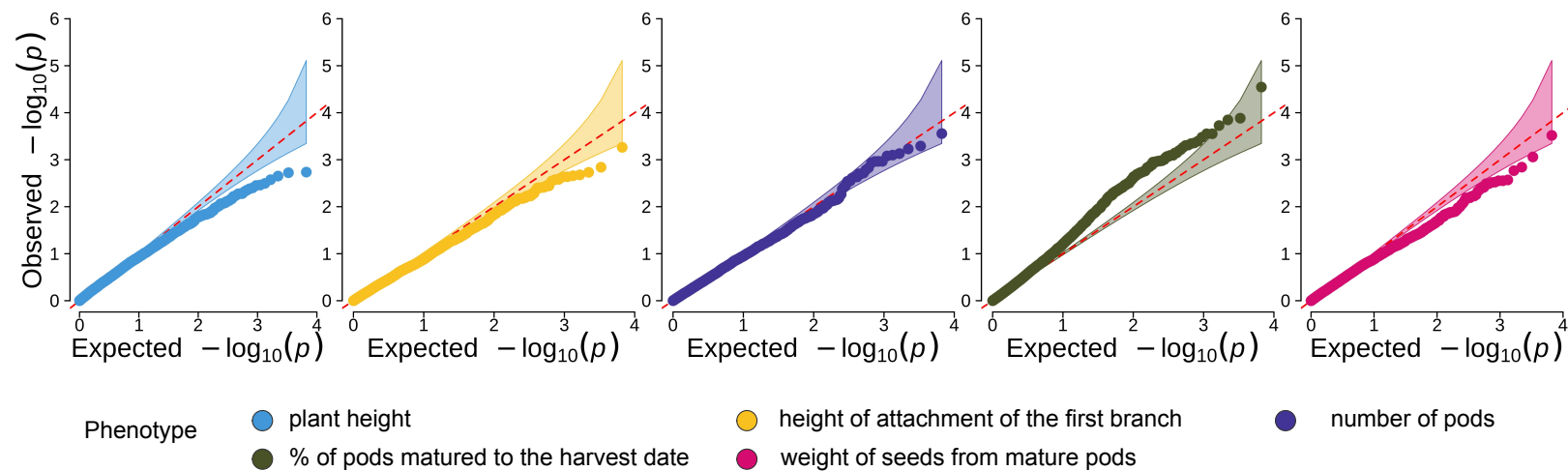**b**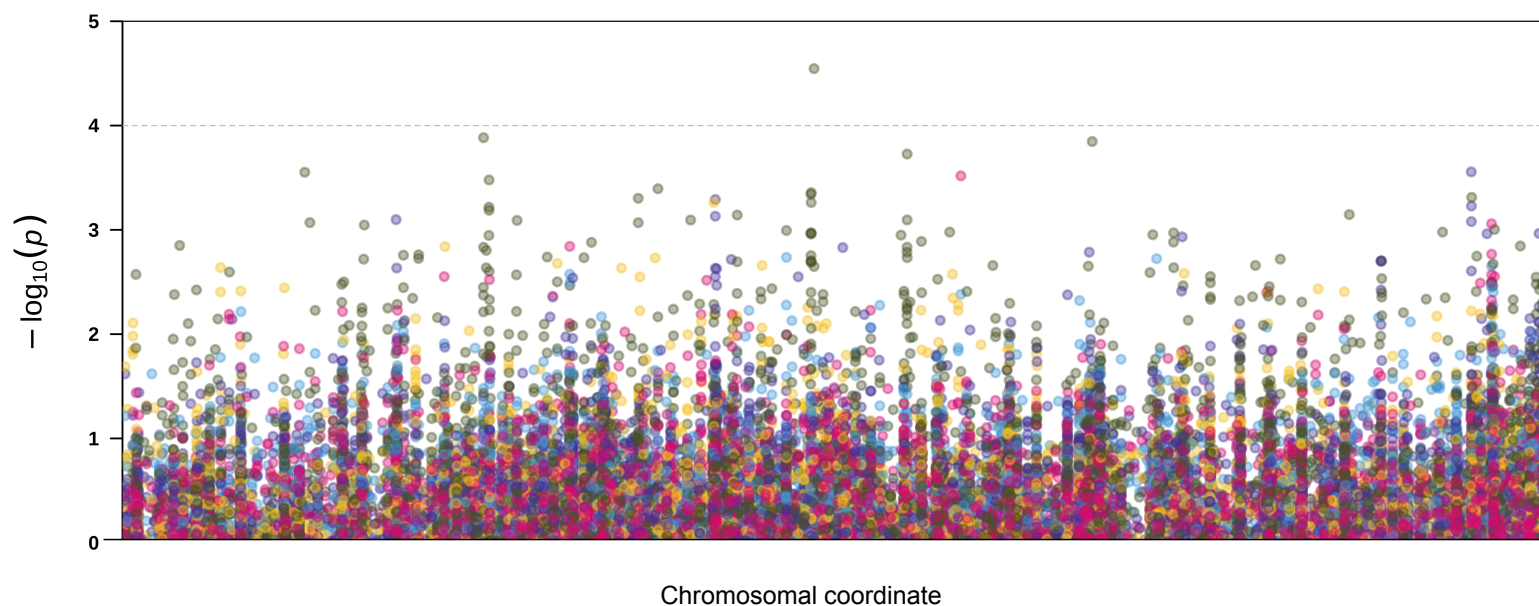

Supplement: Supplementary file 1 [file plants-10-02063-s001.zip › figures/Supplementary Materials Figure S5.pdf]
